# Extended Dynamic Model for the UR16e 6-Degree-of-Freedom Robotic Manipulator

**DOI:** 10.3390/s25247532

**Published:** 2025-12-11

**Authors:** John Kern, Luis Donoso, Claudio Urrea, Guillermo González

**Affiliations:** Electrical Engineering Department, Faculty of Engineering, University of Santiago of Chile, Las Sophoras 165, Estación Central, Santiago 9170020, Chile; luis.donoso@usach.cl (L.D.); claudio.urrea@usach.cl (C.U.); guillermo.gonzalez.r@usach.cl (G.G.)

**Keywords:** extended analytical dynamic model, multibody physical model, Simscape Multibody, actuators, nonlinear system, PD computed torque control

## Abstract

This study develops and validates an Extended Analytical Dynamic Model (EADM) of the UR16e 6-Degree-of-Freedom (DoF) industrial robot, incorporating actuator dynamics and a friction model to address the lack of dynamic information provided by the manufacturer. A two-stage validation methodology is proposed using a Multibody Physical Model (MPM) developed in MATLAB^®^ R2024b/Simscape Multibody^TM^ as a reference. In the first stage, the Analytical Dynamic Model (ADM) without actuators or friction is evaluated by comparing its inverse dynamics torque with the torque required by the MPM under identical joint references. In the second stage, the EADM and the MPM are tested under a Proportional-Derivative Computed Torque Control (PD-CTC) scheme using Cartesian trajectories, comparing joint torques and positions. The methodology incorporates torque-level validation, a demanding criterion since torque is determined by the dynamic formulation, whereas position may be influenced by closed-loop control. The results show small torque errors in the first stage ($e_{\tau }$ in the range of $10^{-17}$ to $10^{-13}$ Nm) and bounded position and torque errors in the second stage ($e_{q}\leq 4\times 10^{-4}$ rad; $e_{\tau }\;\leq 0.4$ Nm in $q_{1}$–$q_{3}$ and $e_{\tau }\leq 0.05$ Nm in $q_{4}$–$q_{6}$). The methodology provides a systematic validation framework and demonstrates that the EADM accurately matches the MPM’s dynamic behavior.

## 1. Introduction

Robotic manipulators have been widely used in industrial applications due to their stability, efficiency, practicality, and safety in automated processes [1,2]. One of the main challenges in the field of robotics lies in the design of advanced control systems capable of ensuring accurate trajectory tracking for reliable task execution [3]. An accurate dynamic model is essential for describing the relationship between joint torques and manipulator motion (position, velocity, and acceleration), for designing high-performance control systems [4,5,6], and for evaluating the performance of controllers in simulation before deployment on a real robot.

Collaborative robots have gained significant relevance in recent years due to their high productivity, flexibility, reduced cost, and inherent safety in human–robot shared environments [7]. However, they differ from traditional industrial manipulators in a fundamental aspect: collaborative robots typically exhibit lower structural stiffness as a result of their lightweight mechanical design, the widespread use of harmonic drive gearboxes—which introduce additional joint elasticity—and the safety requirements associated with human–robot interaction [8]. This reduced stiffness may lead to elastic deformations and end-effector deflections under external loads, payload variations, or contact forces, resulting in positioning errors and vibrations [7,8,9].

Several studies [7,8,10] address these challenges through force-control strategies based on inner/outer loop architectures, in which the inner loop operates in position or velocity, while the outer loop regulates the interaction force. Such strategies are essential in contact-based applications, such as sanding, polishing, and milling. Beyond intended contact scenarios, unexpected collision events must also be handled safely. The approach presented in [11] proposes a unified collision-event pipeline based on monitoring dynamic quantities—such as total energy and generalized momentum—using only proprioceptive sensors. This method is applicable to both rigid and flexible-joint manipulators; in the latter, the motor–link coupling torque acts internally rather than on the environment, enabling collision detection from link-side dynamics.

The dynamics of robotic manipulators are highly nonlinear and coupled due to the complex interactions between the joints and the motion of the system. Two common formulations for describing robot dynamics are the Euler–Lagrange and the Newton–Euler approaches. Both have been extensively adopted in the literature due to their capability to model the physical behavior of manipulators [2,12,13]. These formulations are fundamental to the design of advanced control techniques, as they allow modeling the dynamic relationships among the joint variables.

Nevertheless, many studies simplify the model by considering only the dynamics of the rigid links, neglecting the dynamic effects of the actuators [4,14]. This simplification reduces the realism and accuracy of dynamic models because physical constraints—such as maximum motor torques, gear ratios, and maximum speeds—are not fully represented, thereby affecting the design of advanced model-based controllers [15,16,17]. Therefore, incorporating actuator dynamics leads to an extended dynamic model that more accurately represents the behavior of the complete system.

Since commercial collaborative robots—such as those manufactured by Universal Robots A/S (Odense, Denmark)—do not provide complete dynamic information, multiple studies rely on parameter-identification methods. Deterministic approaches based on least-squares techniques (LLSE/WLSE) have been employed to estimate link and friction parameters [18], and the effects of temperature and mounting configuration on friction-related nonlinear parameters have also been analyzed [19], although the resulting point estimates remain sensitive to noise and do not quantify parameter uncertainty. Other works approximate rigid-body inertias using geometric simplifications and identify actuator parameters using LLSE [20]. More recently, Bayesian learning frameworks have been proposed to estimate dynamic parameters while quantifying uncertainty and enforcing physical feasibility of the parameters through prior information [21].

Beyond parameter identification, several studies validate dynamic models by comparing analytical formulations against Multibody Physical Models (MPMs), particularly when experimental validation is not feasible. In [22], the authors developed the model of the ABB IRB 120 6-Degree-of-Freedom (DoF) robot in Simscape Multibody, incorporating friction effects and validating it against its analytical model, with position errors of the order of $10^{-4}$ to $10^{-2}$ rad. In [23], a digital twin of the AT_00011 manipulator was developed in Simscape, including friction and actuator dynamics, and validated against the analytical model with similar position errors for both models. In [24], the ABB IRB 120 was modeled in Simscape and its dynamic response was compared to an analytical model under identical torque inputs, demonstrating a high level of agreement in joint tracking (errors on the order of $10^{-14}$ to $10^{-13}$ rad), although without considering the effects of friction and actuators. In [25], the dynamic model of a 6-DoF robot is developed using screw theory together with a multibody model simulated in the ADAMS software, where the analytical formulation is validated using the kinematic and dynamic quantities obtained from the multibody simulation. However, the study also does not consider friction effects or actuator dynamics. These studies demonstrate the suitability of MPMs as reference models for validating dynamic behavior under the same operating conditions. Additional studies use models developed in Simscape to evaluate control strategies, as in [26], where a 6-DoF mobile robot was modeled and analyzed under a computed-torque control scheme without considering friction. In addition to manipulators, Simscape Multibody has also been employed for the modeling of parallel robots [27,28] and for evaluating advanced control strategies [29,30,31].

Motivated by the need for a complete dynamic representation, the present study develops and validates an Extended Analytical Dynamic Model (EADM) of the UR16e that incorporates actuator dynamics and a comprehensive friction model—elements typically omitted due to the lack of detailed information provided by the manufacturer. A Multibody Physical Model (MPM) developed in MATLAB^®^ R2024b/Simscape Multibody^TM^ is used as a reference to assess the accuracy of the EADM. The validation methodology consists of two stages: first, the Analytical Dynamic Model (ADM) is evaluated without actuator dynamics or friction; in the second stage, the friction and actuators are incorporated and a Proportional-Derivative Computed Torque Control scheme (PD-CTC) is applied, comparing joint-torque and joint-position errors between the EADM and the MPM. In addition, the trajectory-tracking performance is quantified using the Residual Mean Square (RMS) and the Residual Standard Deviation (RSD) indices.

The main contributions of this work include: the development of an EADM for the UR16e that incorporates actuator dynamics and a comprehensive friction model to compensate for the lack of manufacturer data; the proposal of a validation methodology based on an MPM as reference; and a detailed quantitative comparison of joint torques and joint trajectories, establishing a reproducible framework for validating extended dynamic models of robotic manipulators. Furthermore, this work introduces torque-level validation, a more rigorous and informative criterion than evaluations based only on position. While most existing studies validate dynamic models exclusively at the position level, torque-level comparison directly reflects the accuracy of the dynamic formulation and reveals discrepancies that may remain hidden under closed-loop control. Finally, based on the literature review, no prior studies on dynamic modeling of the UR16e—including actuator and friction dynamics—have been identified.

To clarify and reinforce the main contributions, Table 1 presents a comparison with the most relevant related studies. This comparison shows that the simultaneous inclusion of actuator dynamics and a comprehensive friction model, together with a robust validation methodology, results in an EADM that is better suited for evaluating control strategies prior to experimental implementation.

Most of the studies listed in Table 1 omit actuator dynamics, disregard friction effects, or perform validation exclusively at the joint-position level. In contrast, the torque-level validation employed in this work provides greater transparency regarding the dynamic behavior of the system and strengthens the credibility of the proposed extended analytical dynamic model.

Furthermore, this study could be extended to other manipulators distributed by Universal Robots, since they share a similar mechanical structure, provided that their respective physical and dynamic constraints are properly considered. Although practical implementation faces limitations in terms of time and resources, simulation offers significant advantages by allowing the preliminary evaluation of controllers on an extended dynamic model, thereby ensuring system safety. In addition, this research provides useful guidance for researchers and practitioners developing advanced solutions for this type of robotic arm.

This article is organized as follows: Section 2 describes the system under study; Section 3 describes the validation methodology of the EADM; Section 4 presents the simulations and discusses the results; and Section 5 shows the conclusions and future work.

## 2. Description of the System Under Study

This study utilizes MATLAB^®^ R2024b software on an Asus ZenBook Intel laptop (ASUSTek Computer Inc., Taipei, Taiwan) with an Intel Core i7 processor at 2.8 GHz, 16 GB RAM, and an NVIDIA GeForce MX450 graphics card. The robot selected to model the extended dynamics and validate it against the MPM is the UR16e, a collaborative manipulator manufactured by Universal Robots. This model has 6-DoF, an open structure designed for flexible use, multiple orientation possibilities, and a wide range of communication interfaces with external systems. In addition, it has a payload capacity of 16 kg, a reach of 900 mm, and a repeatability of ±0.05 mm [32].

### 2.1. UR16e Kinematic Model

**Figure 1:**
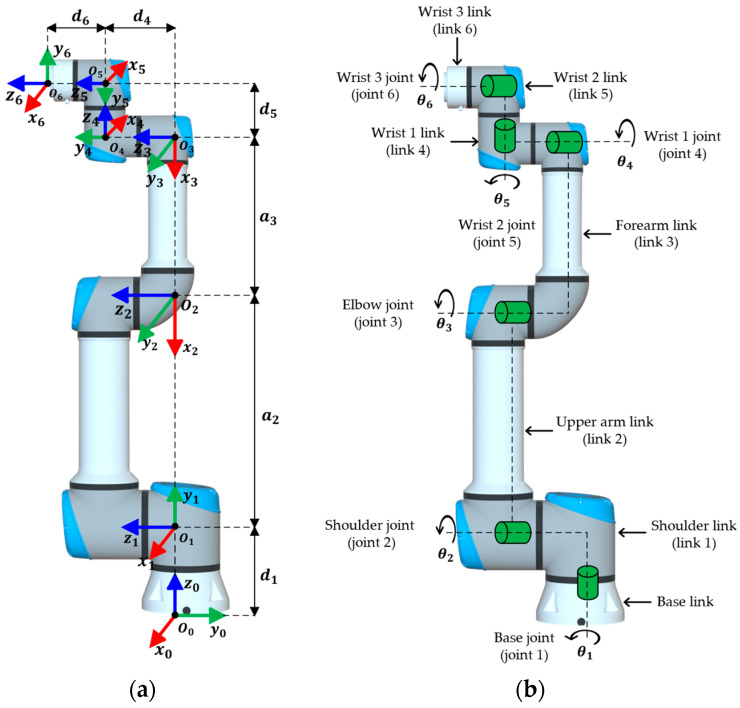
Kinematic structure of the UR16e robot. (**a**) Reference frames according to the D–H convention; (**b**) Joint structure.

The Denavit–Hartenberg (D–H) method is a systematic formulation that allows the description of the geometric relationship between the links of an articulated mechanism and is fundamental to the formulation of a robot’s kinematic model [33]. In this context, Figure 1a shows the symbolic dimensions of the UR16e and the reference frames of each link according to the D–H convention, while Figure 1b shows the location of the joints.

Table 2 presents the D–H parameters [34]. Specifically, i represents the joint number, $\theta_{i}\;$is the angle formed from the $x_{i-1}$ axis to the $x_{i}$ axis measured around the $z_{i-1}$ axis so that two consecutive x-axes are parallel, $d_{i}$ represents the distance along the $z_{i-1}$ axis from the origin $O_{i-1}$ to the intersection of the $x_{i}$ and $z_{i-1}$ axes in order to align consecutive x-axes, $a_{i}$ is the distance along the $x_{i}$ axis from the origin $O_{i}$ to the intersection of the $z_{i}$ y $z_{i-1}$ axes, while $\alpha_{i}$ refers to the angle formed from the $z_{i-1}$ axis to the $z_{i}$ axis measured around the $x_{i}$ axis used to align $O_{i-1}$ with $O_{i}$.

### 2.2. Direct Kinematics

Forward kinematics enables the determination of the position and orientation of the end effector based on the states of the robot’s joint variables [35], with reference to the coordinate system defined at its fixed base. The solution to the forward kinematics problem is based on the geometric parameters and coordinate frames of each link of the robot [36].

The transformation matrix Tii−1 allows relating the coordinate system $O_{i}$ relative to the system $O_{i-1}$, using the parameters obtained from the D–H convention, according to Equation (1):

(1) Tii−1=cosθisinθi00−sinθi·cosαi cosθi·cosαisinαi0 sinθi·sinαi −cosθi·sinαicosαi0aicosθiaisinθidi1.

The general homogeneous transformation matrix, which represents the orientation and position of the end-effector with respect to the robot base coordinate system, is expressed by Equation (2):

(2) T60=T10T21T32T43T54T65=nxoxaxpxnyoyaypynzozazpz0001.

After simplifying the terms of the matrix in (2), the auxiliary notation shown in (3) is adopted.

(3) $$s_{i}=\sin\theta_{i},\;c_{i}=\cos\theta_{i},\;s_{ijk}=\sin\left(\theta_{i}+\theta_{j}+\theta_{k}\right),\;c_{ijk}=\cos\left(\theta_{i}+\theta_{j}+\theta_{k}\right),\;1\leq i,\;j,\;k\leq 6.$$

With this notation, the components of the general homogeneous transformation matrix are given in (4):

(4) $$n_{x}=c_{6}\left(s_{1}s_{5}+c_{234}c_{1}c_{5}\right)-s_{234}c_{1}s_{6},\;n_{y}=-c_{6}\left(c_{1}s_{5}-c_{234}c_{5}s_{1}\right)-s_{234}s_{1}s_{6},\;n_{z}=c_{234}s_{6}+s_{234}c_{5}c_{6},\;o_{x}=-s_{6}\left(s_{1}s_{5}+c_{234}c_{1}c_{5}\right)-s_{234}c_{1}c_{6},\;o_{y}=s_{6}\left(c_{1}s_{5}-c_{234}c_{5}s_{1}\right)-s_{234}c_{6}s_{1},\;o_{z}=c_{234}c_{6}-s_{234}c_{5}s_{6},\;a_{x}=c_{5}s_{1}-c_{234}c_{1}s_{5},\;a_{y}=-c_{1}c_{5}-c_{234}s_{1}s_{5},\;a_{z}=-s_{234}s_{5},\;p_{x}=d_{6}\left(c_{5}s_{1}-c_{234}c_{1}s_{5}\right)+d_{4}s_{1}+a_{2}c_{1}c_{2}+d_{5}s_{234}c_{1}+a_{3}c_{1}c_{2}c_{3}-a_{3}c_{1}s_{2}s_{3},\;p_{y}=a_{2}c_{2}s_{1}-d_{4}c_{1}-d_{6}\left(c_{1}{c_{5}+c}_{234}s_{1}s_{5}\right)+d_{5}s_{234}s_{1}+a_{3}c_{2}c_{3}s_{1}-a_{3}s_{1}s_{2}s_{3},\;p_{z}=d_{1}+a_{3}s_{23}+a_{2}s_{2}-d_{5}\left(c_{23}c_{4}-s_{23}s_{4}\right)-d_{6}s_{5}\left(c_{23}s_{4}+s_{23}c_{4}\right).$$

### 2.3. Inverse Kinematics

The inverse kinematics problem of a robot refers to finding the joint coordinates based on the desired position and orientation of the end effector, with respect to the base coordinate system (i.e., given the general homogeneous transformation matrix T60, the joint variables $\theta_{i}:i\in \left\{1,\cdots ,6\right\}$ are determined). The inverse kinematic solution implemented in this work is based on [37,38]. The coordinate origin vector from the joint axis v to the joint axis u is defined as Pvu, as shown in Equation (5):

(5) Pvu= (Pvu)x (Pvu)y (Pvu)z1.

Since P60 and T60 are known, the vector P50 is calculated using (6):

(6) P50 =P60+ T6000−d61.

**Figure 2:**
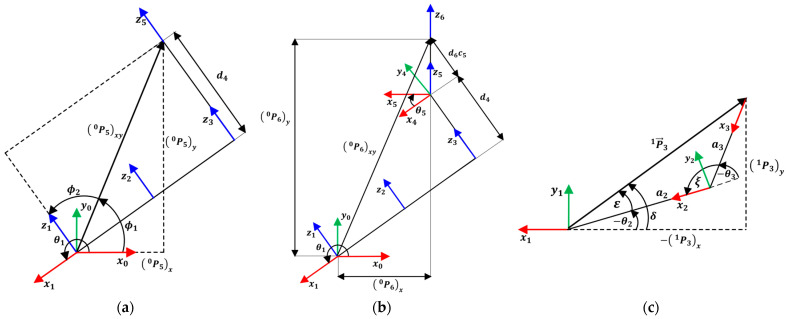
Planar projection of the axis coordinates. (**a**) Planar projection of the fifth coordinate frame referenced to the robot base frame; (**b**) Planar projection of the sixth coordinate frame referenced to the robot base frame; (**c**) Planar projection of the fifth axis to the first axis joint coordinate [37].

From P50 and its components projected onto the plane (P50)x and (P50)y, together with the auxiliary angles $\phi_{1}$ and $\phi_{2}$, $\theta_{1}$ can be determined, as shown is Figure 2a. Equations (7)–(9) define $\theta_{1}$, $\phi_{1}$, and $\phi_{2}$, respectively:

(7) $$\theta_{1}=\phi_{1}+\phi_{2}+\frac{\pi }{2},$$

(8) ϕ1=atan2 P50y P50x,

(9) ϕ2=±cos−1d4 P50x2+ P50y2.

The value of $\theta_{5}$ is determined from the constants $d_{4}$ and $d_{6}$, together with the vector P61 (see Figure 2b), as shown in Equation (10):

(10) θ5=±cos−1 P61z−d4d6.

The vector P61 is obtained from the terms T60 and $P_{6}$, which are known, as given in Equation (11):

(11)  P61 = T60−1 P60.

The transformation matrix T61 can be obtained from T10 and T60 (known), as shown in Equation (12):

(12)  T61 = T10−1 T60=..α0 ..β0 ...0 ...1.

This matrix enables the determination of $\theta_{6}$ through α and β, as indicated in (13):

(13) $$\theta_{6}\;=atan2\left(\frac{-\beta }{\alpha }\right).$$

The joint values $\theta_{3}$ and $\theta_{2}$ are determined using Equations (14) and (15), respectively (see Figure 2c):

(14) θ3=±cos−1P312−a22−a322·a2·a3,

(15) θ2=−atan2P31y−P31x+sin−1a3·sinθ3P31.

The vector P31, required to calculate $\theta_{3}$ and $\theta_{2}$, is given in (16):

(16)  P31= P41+ T410−d401.

The parameter $\theta_{4}$ is determined by Equation (17). For this purpose, the values μ and λ are obtained from the transformation matrix  T43, as given in Equation (18):

(17) $$\theta_{4}\;=atan2\left(\frac{\mu }{\lambda }\right),$$

(18)  T43= T31−1 T41=λμ00 0010 ..00 00.1=cosθ40sinθ40sinθ40−cosθ40010d40001.

### 2.4. Dynamic Model of the UR16e Robot

The dynamics of a robot describe the relationship among the torques, position, velocity, and acceleration of its joints. In other words, the dynamic model of a robotic manipulator aims to describe the relationship between movement and the forces applied to it [39].

For this purpose, the Euler–Lagrange method is employed, which is based on the principle of energy conservation. This formulation is based on the Lagrangian (L) of the system, defined as the difference between the kinetic energy K y and the potential energy P, as shown in Equation (19). Subsequently, L is substituted into the Euler–Lagrange equation, as shown in Equation (20) [12,39]:

(19) $$L\left(q,\;\dot{q}\right)=K\left(q,\dot{q}\right)-P\left(q\right),$$

(20) $$\tau =\frac{d}{dt}\left[\frac{\partial L\left(q,\dot{q}\right)}{\partial \dot{q}}\right]-\frac{\partial L\left(q,\dot{q}\right)}{\partial q}.$$

Thus, the classical dynamic model of a manipulator with n joints can be expressed by Equation (21) [30,40,41]:

(21) $$\tau =M\left(q\right)\ddot{q}+C\left(q,\;\dot{q}\right)\dot{q}+G\left(q\right),\;$$

where $M\left(q\right)\in R^{n\times n}$ is the inertia matrix, $C\left(q,\dot{q}\right)\dot{q}\in R^{n}$ represents the vector of centrifugal and Coriolis forces, and $G\left(q\right)\in R^{n}$ is the gravity vector. The term $\tau \in R^{n}$ corresponds to the vector of generalized forces (forces or torques) applied to each degree of freedom, while $q\in R^{n}$, $\dot{q}\in R^{n}$, and $\ddot{q}\in R^{n}$ represent the joint position, velocity, and acceleration, respectively.

In this model, the friction component is omitted ($F(\dot{q})=0$); its effect is incorporated into the actuator dynamics (see Section 2.6) and, in the MPM, through the Simscape rotational friction block, to prevent duplicated friction effects

The dynamic model of the UR16e robot is determined by employing the physical and dynamic parameters shown in Table 3.

The dynamic model of the 6-DoF UR16e robotic arm is described in Appendix A by Equations (A1)–(A57).

### 2.5. Multibody Physical Model of the UR16e Robot

**Figure 3:**
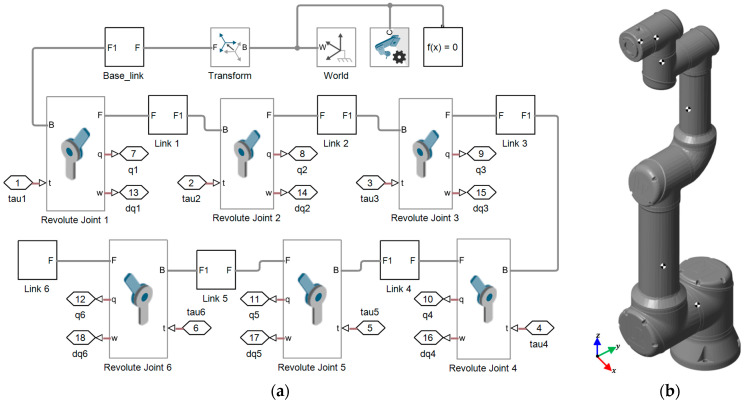
Development in Simscape Multibody. (**a**) Multibody physical model (MPM). (**b**) Generated UR16e robot.

For the development of the MPM, Simscape Multibody is used together with the D–H parameters and the data provided by the robot manufacturer (masses, centers of mass, and inertia tensors). In multibody mechanical systems, Simscape Multibody provides a simulation environment in which rigid bodies and joints are connected within a physical network, and the dynamics are represented by fundamental equations including realistic effects. The MPM and the visualization of the UR16e robot generated using Simscape Multibody are presented in Figure 3.

**Figure 4:**
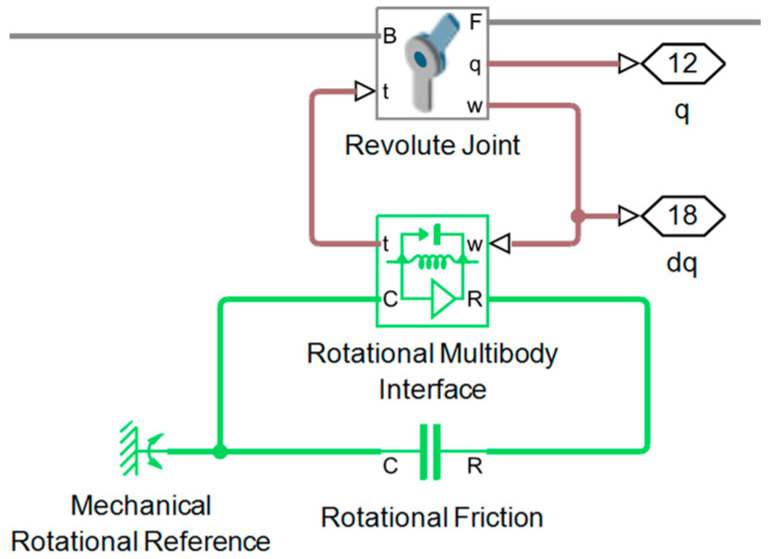
Incorporation of the rotational friction block to a joint in Simscape.

In the MPM, friction is modeled using the Simscape Rotational Friction block and can be incorporated into each joint as shown in Figure 4. In contrast, in the EADM, the friction torque is introduced analytically using the same friction parameters. The friction torque $\tau_{F}$ is a function of the relative velocity ω and represents the sum of the effects of Stribeck, Coulomb, and viscous friction [22,23], given by Equations (22) and (23):

(22) $$\tau_{F}=\sqrt{2e}\left(\tau_{brk}-\tau_{C}\right)exp\left(-{\left(\frac{\omega }{\omega_{St}}\right)}^{2}\right)\frac{\omega }{\omega_{St}}+\tau_{C}\tanh\left(\frac{\omega }{\omega_{Coul}}\right)+f_{v}\omega ,$$

(23) $$\omega_{St}=\omega_{brk}\sqrt{2},\;\omega_{Coul}=\frac{\omega_{brk}}{10},$$

where $\tau_{brk}$ is the breakaway friction torque, $\tau_{C}$ is the Coulomb friction, $f_{v}$ is the viscous friction coefficient, and $\omega_{brk}$ is the breakaway friction velocity, while $\omega_{St}$ and $\omega_{Coul}$ represent the Stribeck velocity threshold and the Coulomb velocity threshold, respectively.

### 2.6. Actuator Dynamics

Universal Robots manipulators, such as the UR16e, are equipped with AC brushless motors manufactured by Kollmorgen, belonging to the AKM series [42]. However, the AKM series used in Universal Robots manipulators are customized, since while the standard models of this series operate at 240 VAC, the motors integrated into the manipulators are powered from a 48 VDC bus [20,43]. Regarding the transmission, studies on the UR5e [20], UR5 [44], and UR10e [45] robots specify that their joints employ Harmonic Drive reducers with a transmission ratio of 100:1. Although no specific technical documentation has been found for the UR16e model, it is presumed that it employs a similar transmission architecture, given the consistent design among the e-series robots.

On the other hand, according to Universal Robots, the UR16e model consists of two size-4 joints (the first two), one size-3 joint (the third joint), and three size-2 joints (the last three). The maximum torques that these joints can exert are 330 Nm, 150 Nm, and 54 Nm, corresponding to sizes 4, 3, and 2, respectively. According to the official documentation, the e-series robots share a modular architecture, which allows the use of the same actuators across different models. In particular, the UR16e employs the same actuators as the UR10e in each joint [46], as shown in Table 4.

Since complete dynamic specifications are not available from the manufacturer, the actuator model is based on the experimentally identified dynamic parameters for the UR10e robot presented in [45] (see Table 5). These parameters allow the actuator behavior to be described using the model proposed in [47], and, in particular, based on the approach in [48] for a joint-space representation, which is the approach adopted in this work.

This formulation adopts the following relationship:

(24) $$q=\;Rq_{m},$$

where $q\in R^{n}$ is the joint position vector, $q_{m}\in R^{n}$ represents the angular position vector of the rotors associated with each joint, and $R\in R^{n\;\times \;n}$ is a diagonal matrix whose diagonal elements are the transmission ratios $r_{i}$ of i-th motor, with $r_{i}<1$.

The dynamics of the actuators driving the manipulator links are given by a set of n equations of the form:

(25) $$J_{m}{\ddot{q}}_{m}+f_{v,m}{\dot{q}}_{m}+\tau_{c,m}+R\tau =K_{m}K_{a}v,$$

where $J_{m}\in R^{n\;\times \;n}$ is the diagonal inertia matrix, whose diagonal elements correspond to the inertia of the motor associated with each joint, $f_{v,m}\in R^{n\;\times \;n}$ is the diagonal viscous friction coefficient matrix, $\tau_{c,m}\in R^{n}$ is the Coulomb friction vector, and $R\tau \in R^{n}$ denotes the vector of torques reflected to the motor side. Moreover, $K_{m}\in R^{n\;\times \;n}$ represents the diagonal torque-constant matrix, $K_{a}$ is the transconductance of the amplifier, and $v\in R^{n}$ is the voltage input vector.

**Figure 5:**
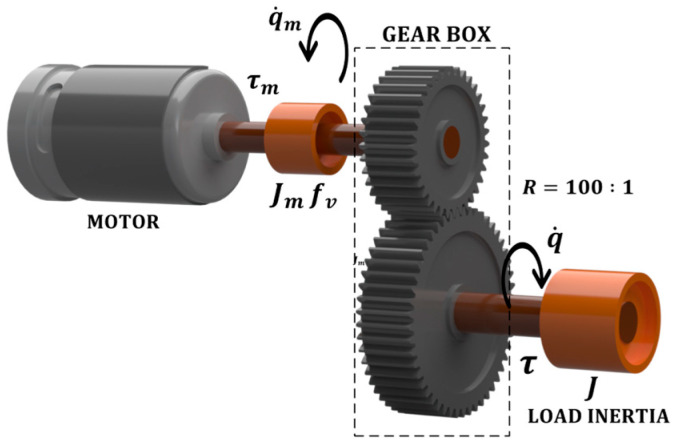
Schematic description of the transmission system of a robot joint.

Figure 5 illustrates a classic schematic description of a robot joint.

Equation (25), corresponding to the dynamics of the actuators, can be incorporated into the dynamic model of the robotic manipulator presented in (21), resulting in an extended description of the robot, which we refer to as the EADM, as shown in Equation (26):

(26) $$\left(M\left(q\right)+\;\frac{J_{m}}{R^{2}}\right)\ddot{q}+\left(\;C\left(q,\dot{q}\right)+\frac{f_{v,m}}{R^{2}}\right)\dot{q}+\left(\;\frac{\tau_{c,m}}{R}\right)+G\left(q\right)=\frac{K_{m}K_{a}v}{R}.$$

In general, the parameters on the motor side and the joint side can be related as follows:

(27) $$J=\frac{J_{m}}{R^{2}},\;f_{v}=\frac{f_{v,m}}{R^{2}},\;\tau_{c}=\frac{\tau_{c,m}}{R},\;K=\frac{K_{m}K_{a}}{R},$$

where J $\in R^{n\;\times \;n}$, $f_{v}\in R^{n\;x\;n}$, $\tau_{c}\in R^{n}$, and $K\in R^{n\;\times \;n}$ denote, respectively, the diagonal inertia matrix, the diagonal viscous friction matrix, the Coulomb friction vector, and the diagonal torque-constant matrix, all expressed on the joint side.

Since the parameters identified experimentally in [45] are expressed on the joint side, it is not necessary to apply the transmission ratios from (26). Therefore, the parameters in (27) are directly employed in the system model, as shown in Equation (28):

(28) $$\left(M\left(q\right)+\;J\right)\ddot{q}+C\left(q,\dot{q}\right)\dot{q}+G\left(q\right)+f_{v}\dot{q}+\tau_{c}=Kv.$$

The friction components in Equation (28) correspond to the classical friction model (Coulomb + viscous). However, to provide greater realism, the rotational friction model is employed, which combines the effects of Stribeck, Coulomb, and viscous friction (see Section 2.5).

**Figure 6:**
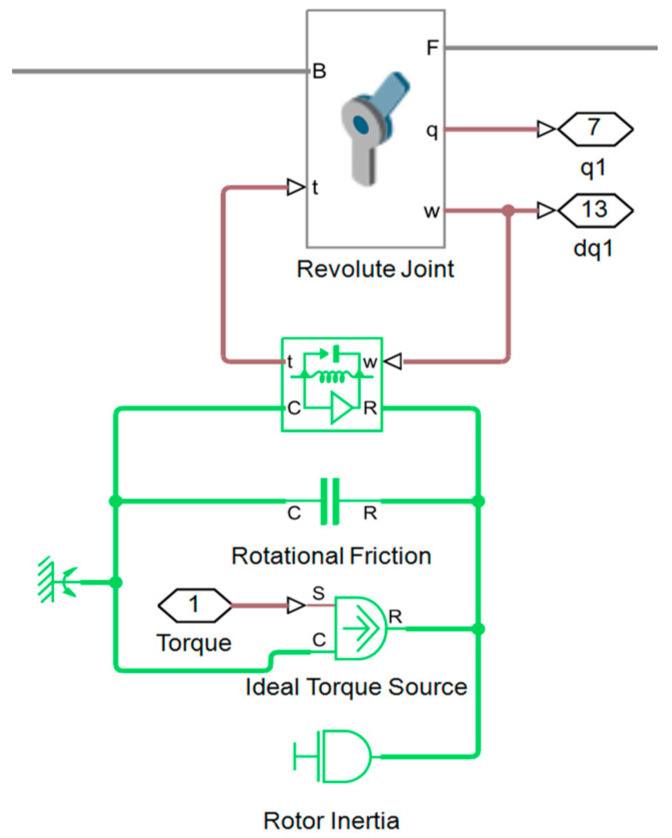
Simscape Multibody revolute joint with torque control, friction and rotor inertia.

In the case of the MPM in Simscape, the rotor inertia and friction are incorporated through the Inertia and Rotational Friction blocks, respectively, as shown in Figure 6. Both blocks are connected to the Revolute Joint block through the Rotational Multibody block, while the control torque is applied using the Ideal Torque Source block in the same circuit. To maintain consistency between the EADM and MPM, the values of the friction and rotor inertia blocks are taken from the same joint-side parameters used in the EADM. The corresponding values are shown in Table 5.

## 3. Validation Methodology

The validation of the EADM is performed by comparing it with the MPM of the robot developed in MATLAB^®^ R2024b/Simulink^®^ R2024b using the Simscape Multibody toolbox. The methodology consists of two stages under the same operating conditions. In the first stage (without actuators), smooth position, velocity, and acceleration references are imposed on the MPM, and the torque required by each joint is compared with the torque determined by the inverse dynamics of the analytical model without actuators (ADM). In the second stage, the actuators are incorporated into both models (EADM and MPM), a PD-CTC controller is applied, and two Cartesian reference trajectories are employed under the same initial conditions, evaluating the joint position and torque errors between both models. In addition, the performance in tracking the reference trajectories is evaluated and quantified using the RMS and RSD indicators.

### 3.1. Stage 1: Validation Without Actuators

The first validation stage considers the evaluation of the torque error between the ADM and the MPM ($e_{\tau }\;=\tau_{MPM}-\tau_{ADM}$), without incorporating the actuator dynamics. For this purpose, smooth position, velocity, and acceleration references are imposed on the MPM, while in the ADM the torque is determined through inverse dynamics, as shown in Equation (29):

(29) $$\tau =M\left(q_{ref}\right){\ddot{q}}_{ref}+C\left(q_{ref},{\;\dot{q}}_{ref}\right){\;\dot{q}}_{ref}+G\left(q_{ref}\right),$$

where $q_{ref}={\left[q_{ref1},\ldots ,\;q_{ref6}\right]}^{T}$ corresponds to the imposed joint trajectories. The joint position references are given in Equation (30):

(30) $$q_{ref1}=0.15\left(1-\cos\left(2\pi t\right)\right),\;q_{ref2}=0.2\left(1-\cos\left(2\pi t\right)\right),\;q_{ref3}=0.3\left(1-\cos\left(2\pi t\right)\right),\;q_{ref4}=0.25\left(1-\cos\left(2\pi t\right)\right),\;q_{ref5}=0.1\left(1-\cos\left(2\pi t\right)\right),\;q_{ref6}=0.5\left(1-\cos\left(2\pi t\right)\right).$$

While ${\;\dot{q}}_{ref}$ and ${\ddot{q}}_{ref}$ are obtained from the first and second derivatives of $q_{ref}$, they represent the reference joint velocity and acceleration, respectively.

**Figure 7:**
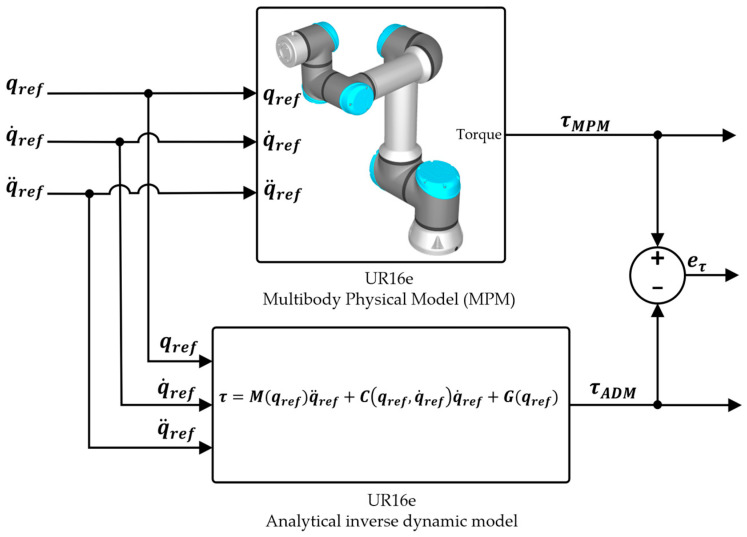
Simulation diagram for output torque comparison between the ADM and the MPM.

The diagram used for the simulation of both models is shown in Figure 7, with a simulation time set to 3 s.

### 3.2. Stage 2: Validation with Actuators

In this stage, the EADM is validated against the MPM of the UR16e, with the actuators incorporated (as described in Section 2.5). For this purpose, a PD-CTC controller and two Cartesian reference trajectories (A and B) are employed under the same initial conditions, evaluating the error of the joint trajectories ($e_{q}\;=q_{MPM}-q_{EADM}$) and control torques ($e_{\tau }\;=\tau_{MPM}-\tau_{EADM}$) produced by both models. In addition, the tracking performance of the reference trajectories is evaluated and quantified using the RMS and RSD indicators.

#### 3.2.1. Proportional-Derivative Computed Torque Control

Computed torque control is a nonlinear control method based on the dynamic model of the manipulator, which represents a special application of feedback linearization in nonlinear systems. Its effectiveness is optimal when robot arm parameters are accurately known. The control torque generated by this method is described in Equation (31) [49,50]:

(31) $$\tau =\hat{M}\left(q\right)\left({\ddot{q}}_{d}+K_{v}\dot{e}+K_{p}e\right)+\hat{C}\left(q,\;\dot{q}\right)\dot{q}+\hat{G}\left(q\right),$$

where $\hat{M}\in R^{n\;x\;n}$ is the estimate of the inertia matrix, $\hat{C}\left(q,\;\dot{q}\right)\dot{q}\in R^{n}$ is the estimate of the centrifugal and Coriolis force vector, and $\hat{G}\;{\in R}^{n}$ is the estimate of the gravity vector. The term ${\ddot{q}}_{d}\in R^{n}$ corresponds to the vector of desired joint accelerations, whereas $K_{v}\in R^{n\;x\;n}$ and $K_{p}\in R^{n\;x\;n}$ are positive definite diagonal matrices representing the derivative and proportional gains, respectively. Finally, $e\;{\in R}^{n}$ y $\overset{˙}{e}\;\in R^{n}$ denote the position error and velocity error vectors, as defined in Equations (32) and (33):

(32) $$e\;=\;q_{d}-q,$$

(33) $$\dot{e}={\dot{q}}_{d}-\dot{q}.$$

If the estimation errors are small, the joint errors can be approximated by a linear equation, as described in Equation (34):

(34) $${\ddot{q}}_{d}+K_{v}\dot{e}+K_{p}e\;\approx \;0.$$

**Figure 8:**
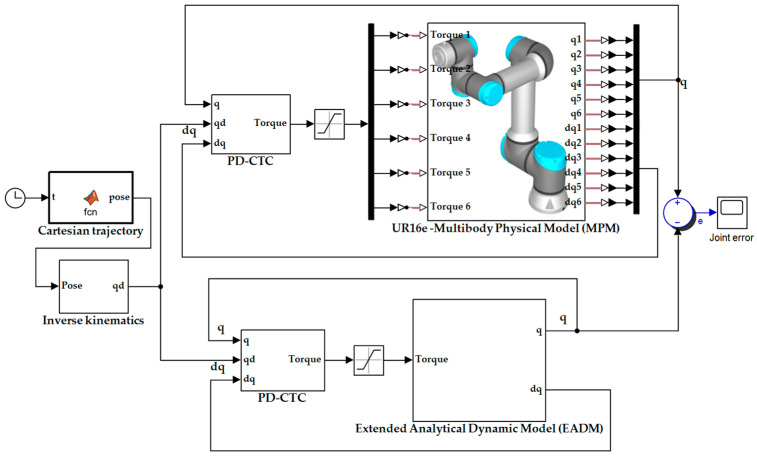
PD-CTC scheme applied to the EADM and the MPM.

The proposed computed torque control method is applied to both models. The actuator dynamics are included in the UR16e robot model (EADM and MPM) in order to simulate the system as close to reality as possible. Figure 8 shows the diagram used in both models.

The gains employed in the PD-CTC controller for both the EADM and MPM are identical, and their values are shown in Table 6.

The tuning of the CT-PD controller follows the gain-selection methodology reported in [51,52]. The procedure uses the decoupled system representation, where step-type inputs are applied to each joint and the controller gains are iteratively adjusted to achieve a fast response, low overshoot, and reduced steady-state error.

#### 3.2.2. Cartesian Trajectories

**Figure 9:**
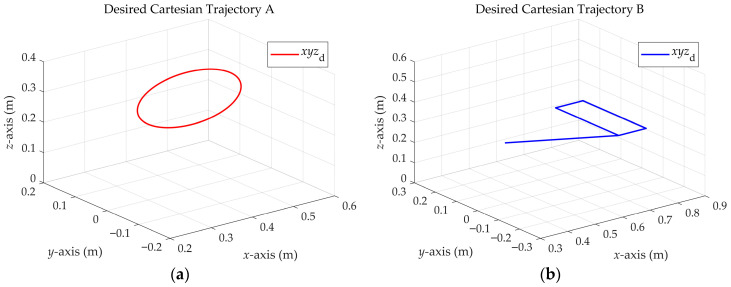
Trajectories in Cartesian space. (**a**) Trajectory A; (**b**) Trajectory B.

To validate the EADM, two Cartesian trajectories are evaluated. Trajectory A corresponds to a circular trajectory that oscillates in height following a cosine function in the z-axis, whose mathematical formulation is given by Equation (35) and illustrated in Figure 9a:

(35) $$\left\{\begin{array}{c} x\;=\;x_{c}+rcos\left(\frac{2\pi }{T}t\right),\\ y\;=\;y_{c}+rsen\left(\frac{2\pi }{T}t\right),\\ z\;=\;z_{c}+Acos\left(\frac{2\pi }{T}t\right), \end{array}\right.$$

where $x_{c}$, $y_{c}$, and $z_{c}$ correspond to the coordinates of the center of the circle, T is the simulation period defined as 8 s, and A corresponds to the oscillation along the z-axis with respect to the coordinate $z_{c}$.

Trajectory B is composed of straight-line segments and vertices, as shown in Figure 9b. This type of trajectory is generally challenging for controllers, since it may cause abrupt changes in velocity and acceleration, inducing vibrations or undesired motions. For its design, the Polynomial Trajectory block in MATLAB^®^ R2024b/Simulink^®^ R2024b was employed, which takes the waypoints and the time to move from one waypoint to another as input. The simulation time for this trajectory is set to 10 s, and the waypoints are defined at the time instants [0, 2, 4, 6, 8, 10].

#### 3.2.3. Performance Indexes

The indexes considered to quantitatively evaluate the controller performance in both models (EADM and MPM) in tracking the desired trajectories are the Residual Mean Square (RMS) and the Residual Standard Deviation (RSD). Both are defined mathematically through Equations (36) and (37), respectively [29,53]:

(36) $$RMS\;=\;\sqrt{\frac{1}{n}\sum_{i=1}^{n}{\left(o_{i}-p_{i}\right)}^{2}},$$

(37) $$RSD=\sqrt{\frac{\sum_{i=1}^{n}{\left(o_{i}-p_{i}\right)}^{2}}{\sum_{i=1}^{n}{o_{i}}^{2}}},$$

where n is the total number of observations, $o_{i}$ corresponds to the observed values, and $p_{i}$ refers to the predicted values.

## 4. Results and Discussion

This section presents the validation results of the EADM compared with the MPM. The validation methodology is described in detail in Section 3.

### 4.1. Stage 1

**Figure 10:**
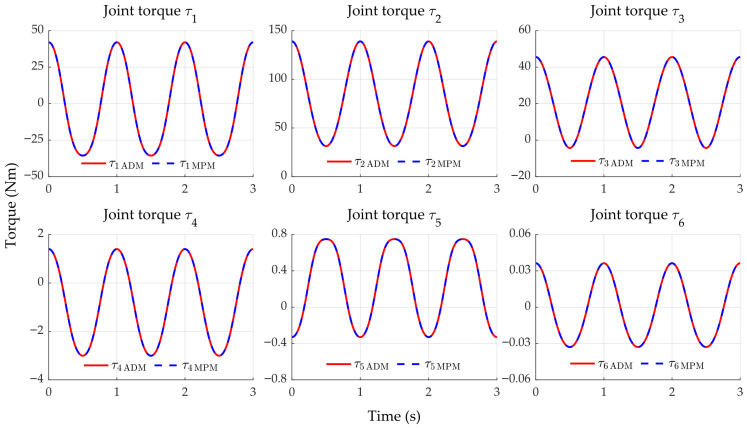
Torque signals produced by the ADM and the MPM.

**Figure 11:**
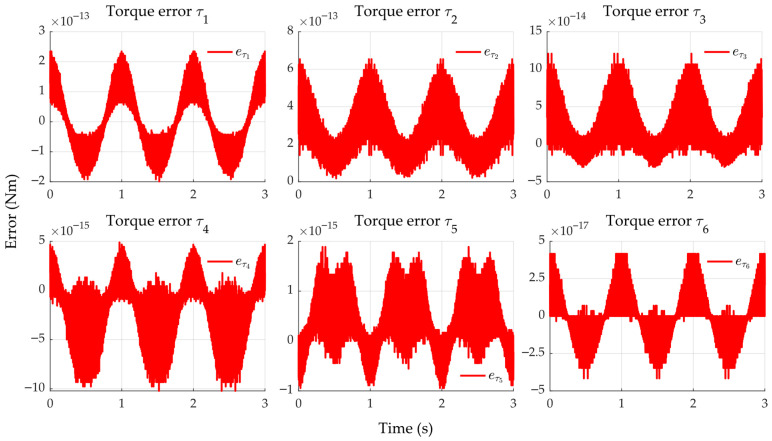
Torque errors between the ADM and the MPM.

By employing smooth position, velocity, and acceleration references in the MPM and computing the torque through the inverse dynamics of the ADM (both without incorporating the actuators), Figure 10 shows that the torque responses closely match and satisfy the maximum joint torques specified by the manufacturer (see Table 4). The torque error between models, $e_{\tau }=\tau_{MPM}-\tau_{ADM}$, remains in the range of $10^{-17}$ to $10^{-13}$ Nm, as shown in Figure 11. This result confirms that the ADM and MPM are equivalent when the actuator dynamics are not considered.

### 4.2. Stage 2

This section presents the validation results of the EADM against the MPM when incorporating the actuators (see Section 3.2). Both models are compared through the error between the joint responses and the control torques, under a PD-CTC controller and two Cartesian trajectories (A and B).

**Figure 12:**
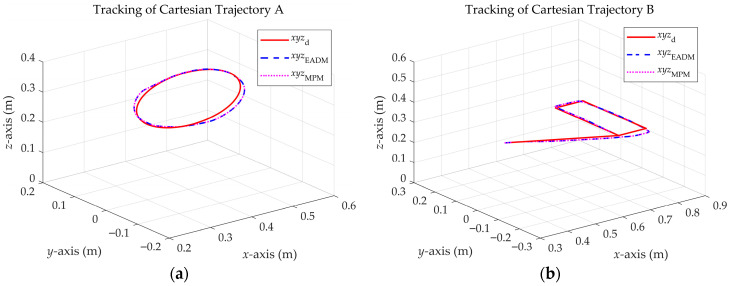
Cartesian trajectory tracking with a PD-CTC controller for the EADM and MPM. (**a**) Trajectory A. (**b**) Trajectory B.

Both models closely match in tracking trajectories A and B, as shown in Figure 12a and Figure 12b, respectively.

**Figure 13:**
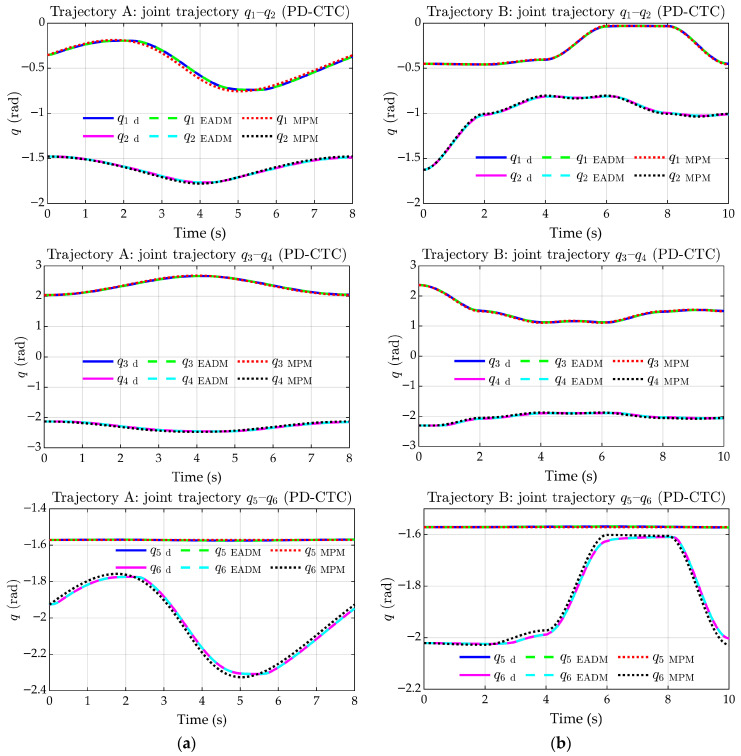
Tracking of joint trajectories. (**a**) Trajectory A. (**b**) Trajectory B.

Figure 13a,b illustrate joint coordinate tracking for Cartesian trajectories A and B, respectively. According to these plots, it can be observed that both models show similar tracking in each trajectory. Joints $q_{1}$, $q_{2}$, $q_{3}$, and $q_{5}$ exhibit negligible deviations, while $q_{4}$ and $q_{6}$ show slight discrepancies with respect to the references.

**Figure 14:**
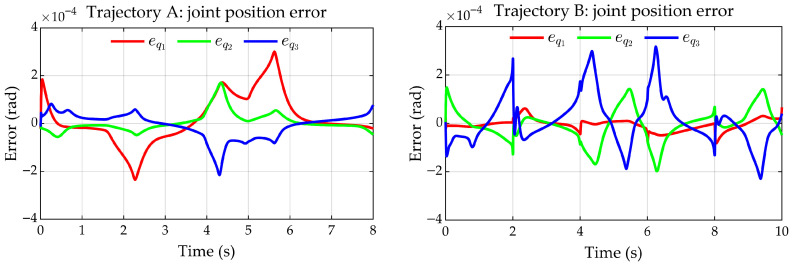

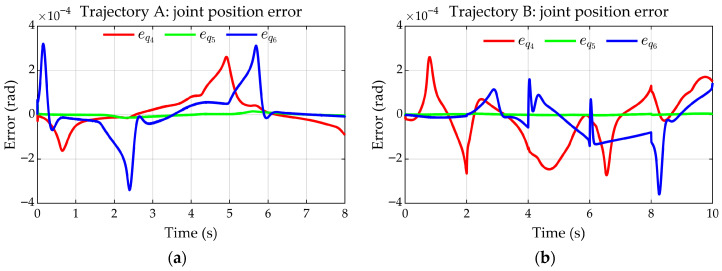
Joint errors between the EADM and the MPM. (**a**) Trajectory A. (**b**) Trajectory B.

The joint error $e_{q}=q_{MPM}-q_{EADM}$ remains within the range of $-3\times 10^{-4}$ to $3\times 10^{-4}$ rad for trajectory A (Figure 14a), whereas for trajectory B it is approximately within the range of $-4\times 10^{-4}$ to $3\times 10^{-4}$ rad (Figure 14b).

**Figure 15:**
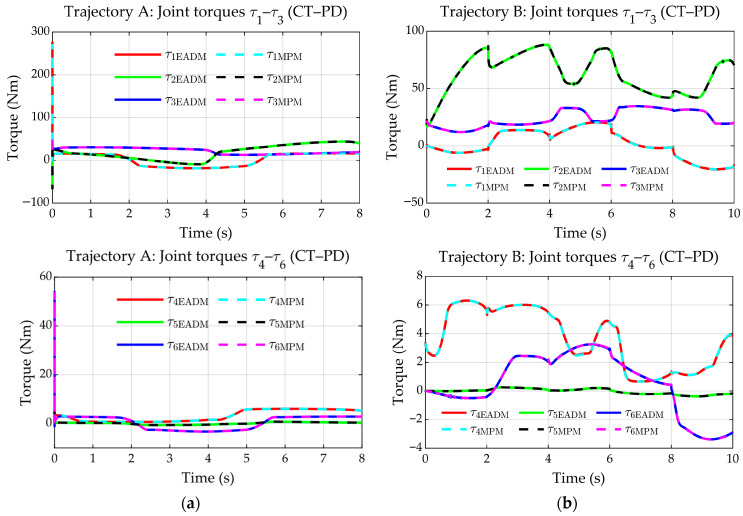
Control torques. (**a**) Trajectory A. (**b**) Trajectory B.

Figure 15a,b show the control torques exerted at each joint for the EADM and MPM in both trajectories (A and B). According to these plots, it can be observed that the torques in both models match and remain within the limits specified by the manufacturer (see Table 4). In trajectory A, an initial peak is observed, associated with the robot starting from rest, i.e., overcoming inertia and friction. In trajectory B, slight torque peaks are identified when the direction changes abruptly at the vertices, particularly affecting $q_{1}$–$q_{3}$, which are the joints that exert the greatest effort.

**Figure 16:**
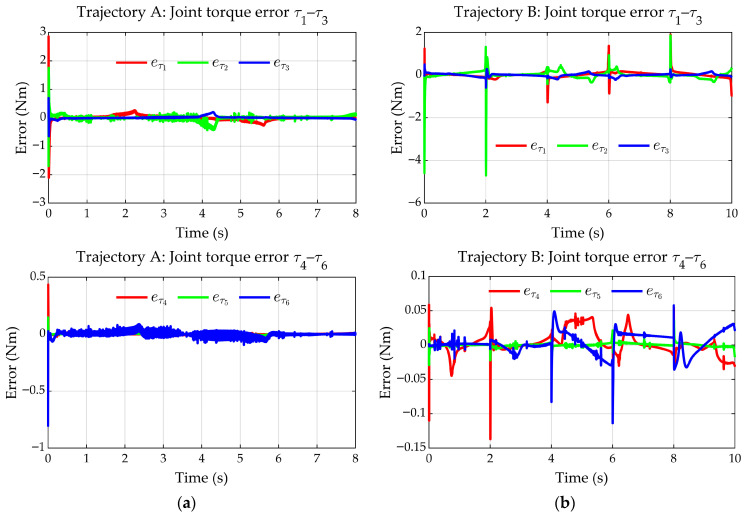
Joint torque errors between the EADM and the MPM. (**a**) Trajectory A. (**b**) Trajectory B.

Figure 16a,b show the torque errors, defined as $e_{\tau }=\tau_{MPM}-\tau_{EADM}$, between the EADM and the MPM. For trajectory A, the torque errors are in the range of −0.4 to 0.3 Nm in $q_{1}$–$q_{3}$ and −0.05 to 0.01 Nm in $q_{4}$–$q_{6}$. However, an initial peak is observed, attributed to numerical errors generated during the calculation of the derivatives required by the PD-CTC controller, which coincides with the initial torque peak (see Figure 15a).

For trajectory B, the torque errors are in the range of −0.3 to 0.4 Nm in $q_{1}$–$q_{3}$ and around ±0.04 Nm in $q_{4}$–$q_{6}$. Peaks are also observed which, as in the previous case, are associated with numerical errors and abrupt direction changes in the trajectory, coinciding in time with the torque peaks (see Figure 15b).

Table 7 and Table 8 present the RMS and RSD performance indices for the tracking of joint trajectories with the same PD-CTC controller applied to the EADM and the MPM, using trajectories A and B, respectively. The results show that both models exhibit values that closely match for each joint, with differences only in the last significant digit. This result, together with the low discrepancy between models observed in the torque and joint trajectory errors, confirms the equivalence between the EADM and the MPM. Therefore, the extended analytical dynamic model is validated.

## 5. Conclusions and Future Work

The main objective of this study was to present and validate the EADM of the UR16e robot against an MPM developed in Simscape Multibody. The validation results of the first stage show that the torques produced by the inverse dynamics of the analytical model match the torques required by the MPM, with $e_{\tau }$ in the range of $10^{-17}$ to $10^{-13}$ Nm, indicating that both models are closely equivalent when the friction and inertia of the actuators are not considered.

In the second validation stage, the actuators are incorporated and a PD-CTC controller is used in both models. The discrepancy between them remains bounded in both reference trajectories, with $e_{q}\leq 4\times 10^{-4}$ rad, $e_{\tau }\leq 0.4$ Nm in $q_{1}$–$q_{3}$, and $e_{\tau }\leq 0.05$ Nm in $q_{4}$–$q_{6}$. These values are consistent with the discrepancies localized at the peaks produced by the startup or by the abrupt direction changes present in the trajectories (especially in trajectory B), and not by recurrent errors of the model.

These findings demonstrate that the EADM reproduces the behavior of the MPM with high fidelity, which allows both models to be used interchangeably. This is of great value for the design and evaluation of controllers in safe environments prior to experimental or real applications. Moreover, this simulation work can significantly reduce the time and cost of research and development. Furthermore, the EADM can be adapted to other UR manipulator models with the same structure by adjusting physical and friction parameters.

Although the present study develops and validates the extended rigid-body dynamic model of the UR16e without interaction from external forces or payload, future challenges of this project will require the manipulator to perform tasks that involve external or contact forces, such as the tightening of hexagonal fasteners. In such scenarios, the robot may experience additional deflections at the joints and at the end-effector due to its low structural stiffness. Therefore, future work will consider the effects of structural elasticity and deflections, as well as force-control strategies based on inner/outer loop architectures —where the inner loop operates in position or velocity, while the outer loop regulates the force and provides the corresponding reference to the inner loop—which have proven effective in compensating for these effects in collaborative manipulators [8,10].

**Figure 17:**
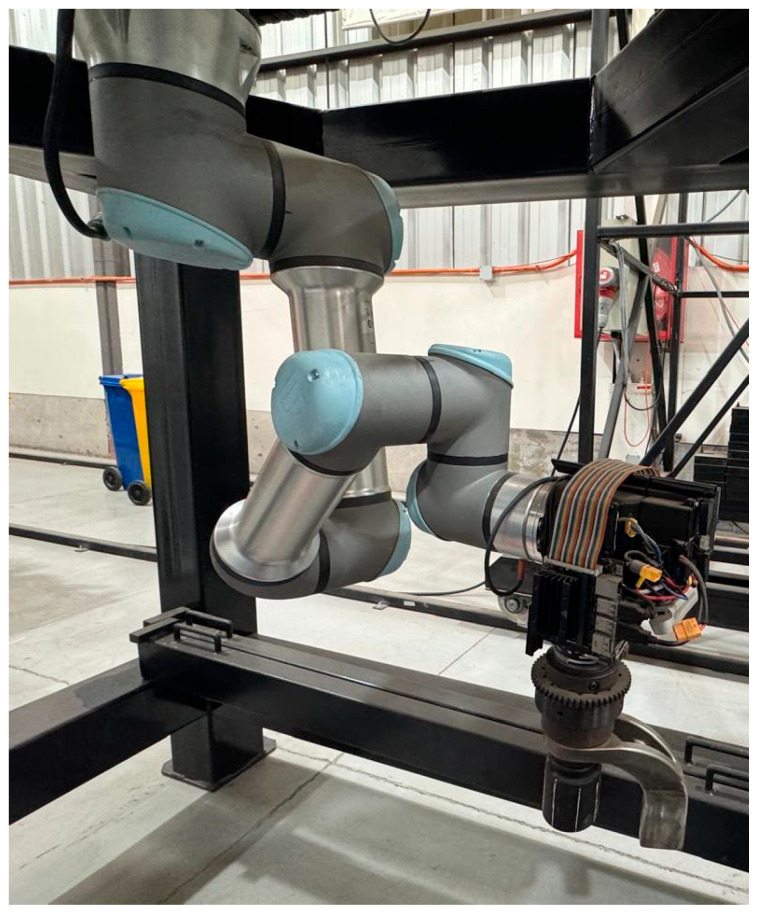
UR16e robot equipped with torque tool.

For future work, this research represents the first stage of a broader project aimed at applying this robotic manipulator to real maintenance tasks in mining, specifically to the tightening of hexagonal fasteners during the replacement of wheels on mining trucks. To this end, it is planned to integrate a torque tool into the end-effector and to develop a computer vision and machine learning algorithm to detect the fasteners and guide the robot, whose output will feed into the robot’s control system. A UR16e robot is currently available at the laboratory level (see Figure 17), which will enable the experimental validation of the EADM and the execution of the planned future work.

## Figures and Tables

**Table 1:** Comparison of Dynamic Modeling and Validation Approaches in Related Works.

Ref.	Robot Model	Model Used	Nonlinear Parameters	Validation Method
[22]	ABB IRB 120	Multibody model included	Actuator dynamics not included	Validation is performed exclusively by analyzing the joint position error between both models
		Analytic model included	Friction model included	
[23]	AT_0001	Multibody model included	Actuator dynamics included	Validation is performed exclusively by analyzing the joint position error between both models
		Analytic model included	Friction model included	
[24]	ABB IRB 120	Multibody model included	Actuator dynamics not included	Validation is performed exclusively by analyzing the joint position error between both models
		Analytic model included	Friction model not included	
[25]	Robotic manipulator of 6-DoF	Multibody model included	Actuator dynamics not included	Validation performed using kinematic and dynamic data provided by the multibody model
		Analytic model included	Friction model not included	

**Table 2:** D–H parameters of the UR16e robot.

Joint	$\theta_{i}$ [rad]	$d_{i}$ [m]	$a_{i}$ [m]	$\alpha_{i}$ [rad]
1	$\theta_{1}$	0.1807	0	π/2
2	$\theta_{2}$	0	−0.4784	0
3	$\theta_{3}$	0	−0.36	0
4	$\theta_{4}$	0.17415	0	π/2
5	$\theta_{5}$	0.11985	0	−π/2
6	$\theta_{6}$	0.11655	0	0

**Table 3:** UR16e dynamic parameters [34].

Dynamics	Mass [kg]	Center of Mass [m]	Inertia Matrix $\left[\mathrm{kg}\;m^{2}\right]$
Link 1	7.369	[0, −0.016, 0.030]	$\left[\begin{array}{ccc} 0.03350 & 0.0000 & 0.0000\\ 0.0000 & 0.0337 & 0.0037\\ 0.0000 & 0.0037 & 0.0210 \end{array}\right]$
Link 2	10.450	[0.302, 0, 0.160]	$\left[\begin{array}{ccc} 0.0280 & -0.0001 & -0.0072\\ -0.0001 & 0.4756 & 0.0000\\ -0.0072 & 0.0000 & 0.4764 \end{array}\right]$
Link 3	4.321	[0.194, 0, 0.065]	$\left[\begin{array}{ccc} 0.0109 & 0.0001 & 0.0101\\ 0.0001 & 0.1206 & 0.0000\\ 0.0101 & 0.0000 & 0.1171 \end{array}\right]$
Link 4	2.180	[0, −0.009, 0.011]	$\left[\begin{array}{ccc} 0.0061 & 0.0000 & 0.0000\\ 0.0000 & 0.0025 & 0.0008\\ 0.0000 & 0.0008 & 0.0058 \end{array}\right]$
Link 5	2.033	[0, 0.018, 0.012]	$\left[\begin{array}{ccc} 0.0039 & 0.0000 & 0.0000\\ 0.0000 & 0.0022 & -0.0005\\ 0.0000 & -0.0005 & 0.0036 \end{array}\right]$
Link 6	0.907	[0, 0, −0.044]	$\left[\begin{array}{ccc} 0.0012 & 0.0000 & 0.0000\\ 0.0000 & 0.0012 & 0.0000\\ 0.0000 & 0.0000 & 0.0008 \end{array}\right]$

**Table 4:** Actuator compatibility and joint torques for UR10e and UR16e robots.

Joint	UR16e	UR10e	Torque [Nm]
1	Size 1	Size 1	330
2	Size 2	Size 2	330
3	Size 3	Size 3	150
4	Size 4	Size 4	54
5	Size 5	Size 5	54
6	Size 6	Size 6	54

**Table 5:** Parameters of actuators and friction (joint side).

Joint	K[Nm/A]	$J[kg\;m^{2}]$	$f_{v}[Nm\;s/rad]$	$\tau_{c}[Nm]$	$\tau_{brk}[Nm]$	$\omega_{brk}[rad/s]$
1	10.00	0.00	21.25	12.54	12.80	0.1
2	10.70	3.74	20.22	13.27	13.5	0.1
3	8.46	0.00	10.38	4.99	5.3	0.1
4	9.0	0.07	3.58	2.00	2.3	0.1
5	9.48	0.23	2.49	2.69	2.85	0.1
6	10.12	0.44	3.03	2.30	2.45	0.1

**Table 6:** PD-CTC controller gains.

Joint	$K_{v}$ [1/seg]	$K_{p}$ $\;[1/{seg}^{2}]$
1	100	800
2	60	400
3	40	1000
4	100	1500
5	20	300
6	20	300

**Table 7:** RMS and RSD performance indices for trajectory A using the PD-CTC controller in the EADM and the MPM.

	RMS		RSD	
	EADM	MPM	EADM	MPM
$q_{1}$	0.022043	0.022046	0.043916	0.043922
$q_{2}$	0.008771	0.008773	0.005431	0.005432
$q_{3}$	0.013679	0.013679	0.005802	0.005802
$q_{4}$	0.022121	0.022117	0.009579	0.009577
$q_{5}$	0.001897	0.001894	0.001208	0.001206
$q_{6}$	0.020170	0.020173	0.009883	0.009884

**Table 8:** RMS and RSD performance indices for trajectory B using the PD-CTC controller in the EADM and the MPM.

	RMS		RSD	
	EADM	MPM	EADM	MPM
$q_{1}$	0.004221	0.004222	0.012861	0.012862
$q_{2}$	0.008892	0.008906	0.008779	0.008793
$q_{3}$	0.014364	0.014375	0.009693	0.009700
$q_{4}$	0.020252	0.020255	0.009986	0.009988
$q_{5}$	0.001288	0.001287	0.000820	0.000819
$q_{6}$	0.016120	0.016139	0.008688	0.008698

## Data Availability

All data are contained within the article.

## Appendix A

To simplify the expressions appearing in the dynamic matrices, compact trigonometric notation is adopted. Table A1 summarizes the notation used. The subscripts may include integer factors, negative signs, or a combination of both, which indicate multiplication or subtraction of the corresponding joint variables, with 1≤i, j, k,l≤6.

**Table A1:** Adopted convention for trigonometric notation.

Notation	Mathematical Meaning
$c_{\left(i\right)}$	$\cos\left(\theta_{i}\right)$
$s_{\left(i\right)}$	$\sin\left(\theta_{i}\right)$
${s_{\left(i\right)}}^{2}$	$\sin{\left(\theta_{i}\right)}^{2}$
$s_{\left(i,j,k\right)}$	$\sin\left(\theta_{i}+\theta_{j}+\theta_{k}\right)$
$c_{\left(i,j,k,l\right)}$	$\cos\left(\theta_{i}+\theta_{j}+\theta_{k}+\theta_{l}\right)$
$s_{\left(2i,-j\right)}$	$\sin\left({2\theta }_{i}-\theta_{j}\right)$

The dynamic model of the 6-DoF UR16e robotic arm is presented in Equations (A1)–(A57): the inertia matrix $M\left(q\right)$ and its components are given in Equations (A1)–(A20); the Coriolis and centrifugal matrix $C\left(q,\dot{q}\right)$ is provided in Equations (A21)–(A51); and the gravity vector $G\left(q\right)$ is detailed in Equations (A52)–(A57).

(A1) $$M=\left[\begin{array}{cccccc} M_{11} & M_{12} & M_{13} & M_{14} & M_{15} & M_{16}\\ M_{21} & M_{22} & M_{23} & M_{24} & M_{25} & M_{26}\\ M_{31} & M_{32} & M_{33} & M_{34} & M_{35} & M_{36}\\ M_{41} & M_{42} & M_{43} & M_{44} & M_{45} & M_{46}\\ M_{51} & M_{52} & M_{53} & M_{54} & M_{55} & M_{56}\\ M_{61} & M_{62} & M_{63} & M_{64} & M_{65} & M_{66} \end{array}\right],$$

(A2) $$M_{26}=M_{36}=M_{46}=M_{62}=M_{63}=M_{64}=0.0080c_{\left(5\right)},$$

(A3) $$M_{25}=M_{52}=c_{\left(5\right)}\left(0.04311s_{\left(3,4\right)}+0.03243s_{\left(4\right)}-0.00987\right),$$

(A4) $$M_{35}=M_{53}=c_{\left(5\right)}\left(0.03247s_{\left(4\right)}-0.00987\right),$$

(A5) $$M_{16}=M_{61}=0.00040c_{\left(2,3,4,5\right)}-0.00040c_{\left(2,3,4,-5\right)},$$

(A6) $$M_{45}=M_{54}=-0.00987c_{\left(5\right)},$$

(A7) $$M_{56}=M_{65}=0,$$

(A8) $$\begin{array}{cc} M_{12}=M_{21}= & 0.05815c_{\left(2,3,4\right)}+0.01623s_{\left(2,3,5\right)}+0.01278c_{\left(2,3,4,-5\right)}+0.00144c_{\left(2,3,4,-25\right)}-0.00144c_{\left(2,3,4,25\right)}\\ & \;+0.02157s_{\left(2,-5\right)}-0.00291c_{\left(2,3,4,5\right)}+0.37065s_{\left(2,3\right)}+0.02157s_{\left(2,5\right)}+0.01623s_{\left(2,3,-5\right)}\\ & \;+0.83928s_{\left(2\right)}, \end{array}$$

(A9) $$\begin{array}{cc} M_{13}=M_{31}= & 0.05815c_{\left(2,3,4\right)}+0.01623s_{\left(2,3,5\right)}+0.01278c_{\left(2,3,4,-5\right)}+0.00144c_{\left(2,3,4,-25\right)}-0.00144c_{\left(2,3,4,25\right)}\\ & \;-0.00291c_{\left(2,3,4,5\right)}+0.37065s_{\left(2,3\right)}+0.01623s_{\left(2,3,-5\right)}, \end{array}$$

(A10) $$M_{14}=M_{41}=0.05815c_{\left(2,3,4\right)}+0.01278c_{\left(2,3,4,-5\right)}+0.00144c_{\left(2,3,4,-25\right)}-0.00144c_{\left(2,3,4,25\right)}-0.00291c_{\left(2,3,4,5\right)},$$

(A11) $$\begin{array}{cc} M_{15}=M_{51} & =0.02157s_{\left(2,-5\right)}-0.01623s_{\left(2,3,5\right)}-0.00291c_{\left(2,3,4,-5\right)}-0.00846c_{\left(2,3,4\right)}-0.01278c_{\left(2,3,4,5\right)}\\ & \;-0.02157s_{\left(2,5\right)}+0.01623s_{\left(2,3,-5\right)}, \end{array}$$

(A12) $$\begin{array}{cc} M_{23}= & M_{32}=1.22493c_{\left(3\right)}-0.24461s_{\left(4\right)}-0.16253c_{\left(3\right)}s_{\left(4\right)}-0.16253c_{\left(4\right)}s_{\left(3\right)}+0.06494c_{\left(4\right)}s_{\left(5\right)}\\ & \;\;\;-0.00576{c_{\left(5\right)}}^{2}-0.04315s_{\left(3\right)}s_{\left(4\right)}s_{\left(5\right)}+0.04315c_{\left(3\right)}c_{\left(4\right)}s_{\left(5\right)}+0.94680, \end{array}$$

(A13) $$\begin{array}{cc} M_{24}= & M_{42}=0.03247c_{\left(4\right)}s_{\left(5\right)}-0.16253c_{\left(3\right)}s_{\left(4\right)}-0.16253c_{\left(4\right)}s_{\left(3\right)}-0.12230s_{\left(4\right)}-0.00576{c_{\left(5\right)}}^{2}\\ & \;\;\;-0.04315s_{\left(3\right)}s_{\left(4\right)}s_{\left(5\right)}+0.04315c_{\left(3\right)}c_{\left(4\right)}s_{\left(5\right)}+0.04704, \end{array}$$

(A14) $$M_{34}=M_{43}=0.03247c_{\left(4\right)}s_{\left(5\right)}-0.12230s_{\left(4\right)}-0.00576{c_{\left(5\right)}}^{2}+0.04704,$$

(A15) $$\begin{array}{cc} M_{11}= & 1.4668c_{\left(22\right)}-0.00072c_{\left(22,23,24,-25\right)}-0.00072c_{\left(22,23,24,25\right)}-0.00493c_{\left(22,23,24,-5\right)}+0.02157s_{\left(3,4,5\right)}\\ & \;\;\;\;\;\;\;\;\;\;+0.02157s_{\left(22,3,4,5\right)}+1.2249c_{\left(22,3\right)}-0.01623s_{\left(4,-5\right)}+0.44616c_{\left(22,23\right)}-0.12231s_{\left(22,23,4\right)}\\ & \;\;\;\;\;\;\;\;\;\;+0.00493c_{\left(22,23,24,5\right)}+0.00144c_{\left(25\right)}-0.01623s_{\left(22,23,4,-5\right)}-0.16253s_{\left(3,4\right)}+0.01623s_{\left(4,5\right)}\\ & \;\;\;\;\;\;\;\;\;\;-0.16253s_{\left(22,3,4\right)}-0.02157s_{\left(3,4,-5\right)}+1.2249c_{\left(3\right)}+0.03141c_{\left(5\right)}-0.02157s_{\left(22,3,4,-5\right)}\\ & \;\;\;\;\;\;\;\;\;\;+0.016623s_{\left(22,23,4,5\right)}-0.12231s_{\left(4\right)}-0.016749c_{\left(22,23,24\right)}+2.4576, \end{array}$$

(A16) $$\begin{array}{cc} M_{22}= & 2.44987c_{\left(3\right)}-0.244987s_{\left(4\right)}-0.32506c_{\left(3\right)}s_{\left(4\right)}-0.32506c_{\left(4\right)}s_{\left(3\right)}+0.06494c_{\left(4\right)}s_{\left(5\right)}+0.00576{c_{\left(5\right)}}^{2}\\ & \;\;\;\;\;\;\;\;\;\;-0.08630s_{\left(3\right)}s_{\left(4\right)}s_{\left(5\right)}+0.08630c_{\left(3\right)}c_{\left(4\right)}s_{\left(5\right)}+3.90911, \end{array}$$

(A17) $$M_{33}=0.06494c_{\left(4\right)}s_{\left(5\right)}-0.24461s_{\left(4\right)}-0.00576{c_{\left(5\right)}}^{2}+0.94680,$$

(A18) $$M_{44}=0.00576{s_{\left(5\right)}}^{2}+0.04128,$$

(A19) $$M_{55}=0.00846,$$

(A20) $$M_{66}=0.00080.$$

(A21) $$C=\left[\begin{array}{cccccc} C_{11} & C_{12} & C_{13} & C_{14} & C_{15} & C_{16}\\ C_{21} & C_{22} & C_{23} & C_{24} & C_{25} & C_{26}\\ C_{31} & C_{32} & C_{33} & C_{34} & C_{35} & C_{36}\\ C_{41} & C_{42} & C_{43} & C_{44} & C_{45} & C_{46}\\ C_{51} & C_{52} & C_{53} & C_{54} & C_{55} & C_{56}\\ C_{61} & C_{62} & C_{63} & C_{64} & C_{65} & C_{66} \end{array}\right],$$

(A22) $$\begin{array}{cc} C_{16}=C_{61}= & 0.0002{\dot{\theta }}_{2}\left(s_{\left(2,3,4,-5\right)}-s_{\left(2,3,4,5\right)}\right)+0.0002{\dot{\theta }}_{3}\left(s_{\left(2,3,4,-5\right)}-s_{\left(2,3,4,5\right)}\right)+0.0002{\dot{\theta }}_{4}\left(s_{\left(2,3,4,-5\right)}-s_{\left(2,3,4,5\right)}\right)\\ & \;-0.0002{\dot{\theta }}_{5}\left(s_{\left(2,3,4,-5\right)}+s_{\left(2,3,4,5\right)}\right), \end{array}$$

(A23) $$C_{26}=C_{36}=C_{46}=-{0.0002\dot{\theta }}_{1}\left(s_{\left(2,3,4,-5\right)}-s_{\left(2,3,4,5\right)}\right)-0.0004{\dot{\theta }}_{5}s_{\left(5\right)},$$

(A24) $$C_{55}=C_{66}=0,$$

(A25) $$C_{62}=C_{63}=C_{64}=0.0002{\dot{\theta }}_{1}\left(s_{\left(2,3,4,-5\right)}-s_{\left(2,3,4,5\right)}\right)-0.0004{\dot{\theta }}_{5}s_{\left(5\right)},$$

(A26) $$\begin{array}{cc} C_{11}= & {\dot{\theta }}_{5}(0.00072\left(s_{\left(22,23,24,25\right)}-s_{\left(22,23,24,-25\right)}\right)-0.00246\left(s_{\left(22,23,24,-5\right)}-s_{\left(22,23,24,5\right)}\right)\\ & \;\;\;\;\;\;\;\;\;\;\;\;\;\;\;\;\;+0.01078\left(c_{\left(3,4,5\right)}+c_{\left(3,4,-5\right)}+c_{\left(22,3,4,5\right)}+c_{\left(22,3,4,-5\right)}\right)\\ & \;\;\;\;\;\;\;\;\;\;\;\;\;\;\;\;\;+0.00811\left(c_{\left(4,5\right)}+c_{\left(4,-5\right)}+c_{\left(22,23,4,5\right)}+c_{\left(22,23,4,-5\right)}\right)-0.00144s_{\left(25\right)}-0.01570s_{\left(5\right)})\\ & \;\;\;\;\;\;\;\;\;\;\;\;\;\;\;\;\;-{\dot{\theta }}_{4}(0.00811\left(c_{\left(4,-5\right)}-c_{\left(4,5\right)}+c_{\left(22,23,4,-5\right)}{-c}_{\left(22,23,4,5\right)}\right)\\ & \;\;\;\;\;\;\;\;\;\;\;\;\;\;\;\;\;-0.00072\left(s_{\left(22,23,24,-25\right)}+s_{\left(22,23,24,25\right)}\right)-0.01078\left(c_{\left(3,4,5\right)}-c_{\left(3,4,-5\right)}+c_{\left(22,3,4,5\right)}-c_{\left(22,3,4,-5\right)}\right)\\ & \;\;\;\;\;\;\;\;\;\;\;\;\;\;\;\;\;+0.00493\left(s_{\left(22,23,24,5\right)}-s_{\left(22,23,24,-5\right)}\right)+0.08126\left(c_{\left(3,4\right)}+c_{\left(22,3,4\right)}\right)\\ & \;\;\;\;\;\;\;\;\;\;\;\;\;\;\;\;\;+0.06115\left(c_{\left(4\right)}+c_{\left(22,23,4\right)}\right)-0.01674s_{\left(22,23,24\right)})\\ & \;\;\;\;\;\;\;\;\;\;\;\;\;\;\;\;\;-{\dot{\theta }}_{3}(0.61247\left(s_{\left(22,3\right)}+s_{\left(3\right)}\right)+0.00493\left(s_{\left(22,23,24,5\right)}-s_{\left(22,23,24,-5\right)}\right)\\ & \;\;\;\;\;\;\;\;\;\;\;\;\;\;\;\;\;-0.00072\left(s_{\left(22,23,24,-25\right)}+s_{\left(22,23,24,25\right)}\right)-0.01078\left(c_{\left(3,4,5\right)}+c_{\left(22,3,4,5\right)}-c_{\left(22,3,4,-5\right)}-c_{\left(3,4,-5\right)}\right)\\ & \;\;\;\;\;\;\;\;\;\;\;\;\;\;\;\;\;+0.01623\left(c_{\left(22,23,4,-5\right)}-c_{\left(22,23,4,5\right)}\right)+0.08126\left(c_{\left(3,4\right)}+c_{\left(22,3,4\right)}\right)-0.01674s_{\left(22,23,24\right)}\\ & \;\;\;\;\;\;\;\;\;\;\;\;\;\;\;\;\;+0.12231c_{\left(22,23,4\right)}+0.44616s_{\left(22,23\right)}){-\dot{\theta }}_{2}(0.00493\left(s_{\left(22,23,24,5\right)}-s_{\left(22,23,24,-5\right)}\right)\\ & \;\;\;\;\;\;\;\;\;\;\;\;\;\;\;\;\;-0.00072\left(s_{\left(22,23,24,25\right)}+s_{\left(22,23,24,-25\right)}\right)+0.02157\left(c_{\left(22,3,4,-5\right)}-c_{\left(22,3,4,5\right)}\right)\\ & \;\;\;\;\;\;\;\;\;\;\;\;\;\;\;\;\;+0.01623\left(c_{\left(22,23,4,-5\right)}+c_{\left(22,23,4,5\right)}\right)-0.01674s_{\left(22,23,24\right)}+1.4668s_{\left(22\right)}+1.2249s_{\left(22,3\right)}\\ & \;\;\;\;\;\;\;\;\;\;\;\;\;\;\;\;\;+0.12231c_{\left(22,23,4\right)}+0.44616s_{\left(22,23\right)}+0.16253c_{\left(22,3,4\right)}), \end{array}$$

(A27) $$\begin{array}{cc} C_{12}= & {\dot{\theta }}_{5}(0.01623\left(c_{\left(2,3,5\right)}-c_{\left(2,3,-5\right)}\right)+0.00144\left(s_{\left(2,3,4,-25\right)}+s_{\left(2,3,4,25\right)}\right)+0.00493\left(s_{\left(2,3,4,-5\right)}-s_{\left(2,3,4,5\right)}\right)\\ & \;\;\;\;\;\;\;\;\;\;+0.02157\left(c_{\left(2,5\right)}-c_{\left(2,-5\right)}\right)-0.00423s_{\left(2,3,4\right)})-{\dot{\theta }}_{6}\left(0.0002\left(s_{\left(2,3,4,5\right)}-s_{\left(2,3,4,-5\right)}\right)\right)\\ & \;\;\;\;\;\;\;\;\;\;+{\dot{\theta }}_{1}(0.00493\left(s_{\left(22,23,24,5\right)}-s_{\left(22,23,24,-5\right)}\right)-0.00072\left(s_{\left(22,23,24,-25\right)}+s_{\left(22,23,24,25\right)}\right)\\ & \;\;\;\;\;\;\;\;\;\;+0.02157\left(c_{\left(22,3,4,-5\right)}-c_{\left(22,3,4,5\right)}\right)+0.01623\left(c_{\left(22,23,4,-5\right)}-c_{\left(22,23,4,5\right)}\right)-0.01674s_{\left(22,23,4,-5\right)}\\ & \;\;\;\;\;\;\;\;\;\;+1.2249s_{\left(22,3\right)}+0.12231c_{\left(22,23,4\right)}+0.44616s_{\left(22,23\right)}+0.16253c_{\left(22,3,4\right)}+1.4668s_{\left(22\right)}), \end{array}$$

(A28) $$\begin{array}{cc} C_{13}= & {\dot{\theta }}_{6}(0.0002(s_{\left(2,3,4,-5\right)}-s_{\left(2,3,4,5\right)})-{\dot{\theta }}_{4}(0.05815s_{\left(2,3,4\right)}+0.01278s_{\left(2,3,4,-5\right)}-0.00144(s_{\left(2,3,4,-25\right)}\\ & \;\;\;\;\;\;\;\;+s_{\left(2,3,4,25\right)})-0.00291s_{\left(2,3,4,5\right)})-{\dot{\theta }}_{1}(0.61247(s_{\left(22,3\right)}+s_{\left(3\right)})+0.00493(s_{\left(22,23,24,5\right)}\\ & \;\;\;\;\;\;\;\;-s_{\left(22,23,24,-5\right)})-0.00072(s_{\left(22,23,24,-25\right)}+s_{\left(22,23,24,25\right)})+0.01078(c_{\left(3,4,-5\right)}+c_{\left(22,3,4,-5\right)}\\ & \;\;\;\;\;\;\;\;-c_{\left(3,4,5\right)}-c_{\left(22,3,4,5\right)})+0.01623(c_{\left(22,23,4,-5\right)}-c_{\left(22,23,4,-5\right)})+0.08126(c_{\left(3,4\right)}+c_{\left(22,3,4\right)})\\ & \;\;\;\;\;\;\;\;+0.12231c_{\left(22,23,4,\right)}+0.44616s_{\left(22,23\right)}+0.01674s_{\left(22,23,24\right)})+{\dot{\theta }}_{2}(0.00144(s_{\left(2,3,4,25\right)}\\ & \;\;\;\;\;\;\;\;+s_{\left(2,3,4,-25\right)})+0.01623(c_{\left(2,3,5\right)}+c_{\left(2,3,-5\right)})+0.37065c_{\left(2,3\right)}+0.00291s_{\left(2,3,4,5\right)}\\ & \;\;\;\;\;\;\;\;-0.01279s_{\left(2,3,4,-5\right)}-0.05815s_{\left(2,3,4\right)})+{\dot{\theta }}_{3}(0.00144(s_{\left(2,3,4,-25\right)}+s_{\left(2,3,4,25\right)})\\ & \;\;\;\;\;\;\;\;+0.01623(c_{\left(2,3,5\right)}+c_{\left(2,3,-5\right)})+0.37065c_{\left(2,3\right)}+0.00291s_{\left(2,3,4,5\right)})-0.01279s_{\left(2,3,4,-5\right)}\\ & \;\;\;\;\;\;\;\;-0.05815s_{\left(2,3,4\right)})+{\dot{\theta }}_{5}(0.00144\left(s_{\left(2,3,4,25\right)}+s_{\left(2,3,4,-5\right)}\right)\\ & \;\;\;\;\;\;\;\;+0.00785\left(s_{\left(2,3,4,-5\right)}+s_{\left(2,3,4,5\right)}+0.00423s_{\left(2,3,4\right)}\right), \end{array}$$

(A29) $$\begin{array}{cc} C_{14}= & {\dot{\theta }}_{6}(0.0002(s_{\left(2,3,4,-5\right)}-s_{\left(2,3,4,5\right)})-{\dot{\theta }}_{2}(0.00144(s_{\left(2,3,4,-25\right)}-s_{\left(2,3,4,25\right)})-0.00291s_{\left(2,3,4,5\right)}\\ & \;\;\;+0.01279s_{\left(2,3,4,-5\right)}+0.05815s_{\left(2,3,4\right)})-{\dot{\theta }}_{3}(0.00144(s_{\left(2,3,4,-25\right)}-s_{\left(2,3,4,25\right)})\\ & \;\;\;+0.01279s_{\left(2,3,4,-5\right)}-0.00291s_{\left(2,3,4,5\right)}+0.05815s_{\left(2,3,4\right)})-{\dot{\theta }}_{4}(0.05815s_{\left(2,3,4\right)}\\ & \;\;\;+0.01279s_{\left(2,3,4,-5\right)}-0.00144(s_{\left(2,3,4,-25\right)}-s_{\left(2,3,4,25\right)})-0.00291s_{\left(2,3,4,5\right)})\\ & \;\;\;-{\dot{\theta }}_{1}(0.00811(c_{\left(4,-5\right)}+c_{\left(22,23,4,-5\right)}{-c}_{\left(22,23,4,5\right)})-0.00072(s_{\left(22,23,24,-25\right)}+s_{\left(22,23,24,25\right)})\\ & \;\;\;+0.01078(c_{\left(22,3,4,-5\right)}+c_{\left(3,4,-5\right)}{-c}_{\left(3,4,5\right)}{-c}_{\left(22,3,4,5\right)})+0.08126(c_{\left(3,4\right)}+c_{\left(22,3,4\right)})\\ & \;\;\;+0.06115(c_{\left(4\right)}+c_{\left(22,23,4\right)})+0.00493(s_{\left(22,23,24,5\right)}-s_{\left(22,23,24,-5\right)})-0.01674s_{\left(22,23,24\right)}\\ & \;\;\;-0.00811c_{\left(4,5\right)})+{\dot{\theta }}_{5}(0.00144(s_{\left(2,3,4,-25\right)}+s_{\left(2,3,4,25\right)})+0.00785(s_{\left(2,3,4,-5\right)}+s_{\left(2,3,4,5\right)})\\ & \;\;\;+0.00423s_{\left(2,3,4\right)}), \end{array}$$

(A30) $$\begin{array}{cc} C_{15}= & {\dot{\theta }}_{1}(0.00811(c_{\left(4,5\right)}+c_{\left(4,-5\right)}+c_{\left(22,23,4,-5\right)}+c_{\left(22,23,4,5\right)})+0.00072(s_{\left(22,23,24,25\right)}-s_{\left(22,23,24,-25\right)})\\ & \;\;\;\;\;\;\;\;+0.01078(c_{\left(3,4,5\right)}+c_{\left(22,3,4,5\right)}+c_{\left(3,4,-5\right)}+c_{\left(22,3,4,-5\right)})-0.00246(s_{\left(22,23,24,-5\right)}+s_{\left(22,23,24,5\right)})\\ & \;\;\;\;\;\;\;\;-0.00144s_{\left(25\right)}-0.01570s_{\left(5\right)})-{\dot{\theta }}_{6}(0.0002(s_{\left(2,3,4,-5\right)}+s_{\left(2,3,4,5\right)}))-{\dot{\theta }}_{5}(0.016236(c_{\left(2,3,5\right)}\\ & \;\;\;\;\;\;\;\;+c_{\left(3,4,-5\right)})+0.02157(c_{\left(2,-5\right)}+c_{\left(2,5\right)})+0.00291s_{\left(2,3,4,-5\right)}-0.01279s_{\left(2,3,4,5\right)})\\ & \;\;\;\;\;\;\;\;+{\dot{\theta }}_{2}(0.00144(s_{\left(2,3,4,-25\right)}+s_{\left(2,3,4,25\right)})+0.00785(s_{\left(2,3,4,-5\right)}+s_{\left(2,3,4,5\right)})+0.00423s_{\left(2,3,4\right)})\\ & \;\;\;\;\;\;\;\;+{\dot{\theta }}_{3}(0.00423s_{\left(2,3,4\right)}+0.00785(s_{\left(2,3,4,-5\right)}+s_{\left(2,3,4,5\right)})+0.00144(s_{\left(2,3,4,25\right)}\\ & \;\;\;\;\;\;\;\;+s_{\left(2,3,4,-25\right)})){\dot{\theta }}_{4}(0.00423s_{\left(2,3,4\right)}+0.00785(s_{\left(2,3,4,-5\right)}+s_{\left(2,3,4,5\right)})+0.00144(s_{\left(2,3,4,25\right)}\\ & \;\;\;\;\;\;\;\;+s_{\left(2,3,4,-25\right)}), \end{array}$$

(A31) $$\begin{array}{cc} C_{21}= & {\dot{\theta }}_{5}(0.016236(c_{\left(2,3,5\right)}-c_{\left(3,4,-5\right)})+0.00144(s_{\left(2,3,4,-25\right)}+s_{\left(2,3,4,25\right)})+0.00493(s_{\left(2,3,4,-5\right)}-s_{\left(2,3,4,5\right)})\\ & \;\;\;\;\;\;\;\;\;\;\;\;+0.02157(c_{\left(2,-5\right)}-c_{\left(2,-5\right)})-0.00423s_{\left(2,3,4\right)})-{\dot{\theta }}_{6}0.0002\left(s_{\left(2,3,4,-5\right)}-s_{\left(2,3,4,5\right)}\right)\\ & \;\;\;\;\;\;\;\;\;\;\;\;+{\dot{\theta }}_{1}(1.4668s_{\left(22\right)}+0.00493(s_{\left(22,23,24,5\right)}-s_{\left(22,23,24,-5\right)})\\ & \;\;\;\;\;\;\;\;\;\;\;\;-0.00072(s_{\left(22,23,24,25\right)}+s_{\left(22,23,24,-25\right)})+0.01623(c_{\left(22,23,4,-5\right)}-c_{\left(22,23,4,5\right)})\\ & \;\;\;\;\;\;\;\;\;\;\;\;+0.02157(c_{\left(22,3,4,-5\right)}-c_{\left(22,3,4,5\right)})-0.01674s_{\left(22,23,24\right)})+1.2249s_{\left(22,3\right)}+0.12231c_{\left(22,23,4\right)}\\ & \;\;\;\;\;\;\;\;\;\;\;\;+0.16253c_{\left(22,3,4\right)}+0.44616s_{\left(22,23\right)}, \end{array}$$

(A32) $$\begin{array}{cc} C_{22}= & -{\dot{\theta }}_{4}(0.12231c_{\left(4\right)}+0.16253(c_{\left(3\right)}c_{\left(4\right)}-s_{\left(3\right)}s_{\left(4\right)})+0.043151({c_{\left(3\right)}c_{\left(4\right)}s}_{\left(5\right)}+{c_{\left(4\right)}s}_{\left(3\right)}s_{\left(5\right)})\\ & \;\;+0.04315({c_{\left(3\right)}s}_{\left(4\right)}s_{\left(5\right)}+{c_{\left(4\right)}s}_{\left(3\right)}s_{\left(5\right)}))\\ & \;\;-{\dot{\theta }}_{3}\left(1.2249s_{\left(3\right)}+0.16253(c_{\left(3\right)}c_{\left(4\right)}-s_{\left(3\right)}s_{\left(4\right)}+0.04315\left({c_{\left(4\right)}s}_{\left(3\right)}s_{\left(5\right)}-{c_{\left(3\right)}s}_{\left(4\right)}s_{\left(5\right)}\right))\right), \end{array}$$

(A33) $$\begin{array}{cc} C_{23}= & {\dot{\theta }}_{5}(0.02157(c_{\left(3,4,5\right)}+c_{\left(3,4,-5\right)})+0.01623(c_{\left(4,-5\right)}+c_{\left(4,5\right)})+0.00288s_{\left(25\right)})-{\dot{\theta }}_{3}(1.2249s_{\left(3\right)}\\ & \;\;\;\;\;\;+0.16253c_{\left(3,4\right)})+0.02157(c_{\left(3,4,-5\right)}-c_{\left(3,4,5\right)})-{\dot{\theta }}_{4}(0.01623(c_{\left(4,-5\right)}-c_{\left(4,5\right)})\\ & \;\;\;\;\;\;+0.02157(c_{\left(3,4,-5\right)}-c_{\left(3,4,5\right)})+0.16253c_{\left(3,4\right)}+0.12231c_{\left(4\right)})-{\dot{\theta }}_{2}(0.16253c_{\left(3,4\right)}\\ & \;\;\;\;\;\;+1.2249s_{\left(3\right)}+0.02157\left(c_{\left(3,4,-5\right)}-c_{\left(3,4,5\right)}\right), \end{array}$$

(A34) $$\begin{array}{cc} C_{24}= & {\dot{\theta }}_{5}(0.04315(c_{\left(3\right)}c_{\left(4\right)}c_{\left(5\right)}-{c_{\left(5\right)}s}_{\left(3\right)}s_{\left(4\right)})+0.00288s_{\left(25\right)}+0.03247c_{\left(4\right)}c_{\left(5\right)})-{\dot{\theta }}_{2}(0.12231c_{\left(4\right)}\\ & \;\;\;\;\;\;+0.03247s_{\left(4\right)}s_{\left(5\right)}+0.16253(c_{\left(3\right)}c_{\left(4\right)}-s_{\left(3\right)}s_{\left(4\right)})+0.04315({c_{\left(3\right)}s}_{\left(4\right)}s_{\left(5\right)}+{c_{\left(4\right)}s}_{\left(3\right)}s_{\left(5\right)})\\ & \;\;\;\;\;\;-{\dot{\theta }}_{3}(0.12231c_{\left(4\right)}+0.16253(c_{\left(3\right)}c_{\left(4\right)}-s_{\left(3\right)}s_{\left(4\right)})+0.03247s_{\left(4\right)}s_{\left(5\right)}+0.04315({c_{\left(3\right)}s}_{\left(4\right)}s_{\left(5\right)}\\ & \;\;\;\;\;\;+{c_{\left(4\right)}s}_{\left(3\right)}s_{\left(5\right)}))-{\dot{\theta }}_{4}(0.12231c_{\left(4\right)}+0.16253(c_{\left(3\right)}c_{\left(4\right)}-s_{\left(3\right)}s_{\left(4\right)})+0.04315({c_{\left(3\right)}s}_{\left(4\right)}s_{\left(5\right)}\\ & \;\;\;\;\;\;+{c_{\left(4\right)}s}_{\left(3\right)}s_{\left(5\right)})+0.03247s_{\left(4\right)}s_{\left(5\right)}), \end{array}$$

(A35) $$\begin{array}{cc} C_{25}= & {\dot{\theta }}_{5}(0.02157(c_{\left(3,4,5\right)}-c_{\left(3,4,-5\right)})+0.01623(c_{\left(4,5\right)}-c_{\left(4,-5\right)})+0.00987s_{\left(5\right)})+{\dot{\theta }}_{1}(0.01623(c_{\left(2,3,5\right)}\\ & \;\;\;\;\;\;\;\;-c_{\left(2,3,-5\right)})+0.00493(s_{\left(2,3,4,-5\right)}-s_{\left(2,3,4,5\right)})+0.00144(s_{\left(2,3,4,-25\right)}+s_{\left(2,3,4,25\right)})\\ & \;\;\;\;\;\;\;\;+0.02157(c_{\left(2,5\right)}-c_{\left(2,-5\right)})-0.00423s_{\left(2,3,4\right)}){-0.0004\dot{\theta }}_{6}s_{\left(2,3,4\right)}+{\dot{\theta }}_{2}(0.02157(c_{\left(3,4,5\right)}\\ & \;\;\;\;\;\;\;\;+c_{\left(3,4,-5\right)})+0.01623(c_{\left(4,5\right)}+c_{\left(4,-5\right)})+0.00288s_{\left(25\right)})+{\dot{\theta }}_{3}(0.01623(c_{\left(4,5\right)}+c_{\left(4,-5\right)})\\ & \;\;\;\;\;\;\;\;+0.02157(c_{\left(3,4,5\right)}+c_{\left(3,4,-5\right)})+0.00288s_{\left(25\right)})+{\dot{\theta }}_{4}(0.02157(c_{\left(3,4,5\right)}+c_{\left(3,4,-5\right)})\\ & \;\;\;\;\;\;\;\;+0.01623(c_{\left(4,5\right)}+c_{\left(4,-5\right)})+0.00288s_{\left(25\right)}), \end{array}$$

(A36) $$\begin{array}{cc} C_{31}= & {\dot{\theta }}_{5}(0.01623(c_{\left(2,3,5\right)}-c_{\left(2,3,-5\right)})+0.00493(s_{\left(2,3,4,-5\right)}-s_{\left(2,3,4,5\right)})+0.00144(s_{\left(2,3,4,-25\right)}-s_{\left(2,3,4,25\right)})\\ & \;\;\;\;\;\;\;\;\;\;-0.00423s_{\left(2,3,4\right)})+{\dot{\theta }}_{1}(0.62147(s_{\left(3\right)}+s_{\left(22,3\right)})+0.00493(s_{\left(22,23,24,5\right)}-s_{\left(22,23,24,-5\right)})\\ & \;\;\;\;\;\;\;\;\;\;-0.00072(s_{\left(22,23,24,-25\right)}+s_{\left(22,23,24,25\right)})-0.01078(c_{\left(2,3,5\right)}+c_{\left(22,3,4,5\right)}-c_{\left(2,3,-5\right)}-c_{\left(22,3,4,-5\right)})\\ & \;\;\;\;\;\;\;\;\;\;+0.01623(c_{\left(22,23,4,-5\right)}-c_{\left(22,23,4,5\right)})+0.08126(c_{\left(3,4\right)}+c_{\left(22,3,4\right)})+0.01674s_{\left(22,23,24\right)}\\ & \;\;\;\;\;\;\;\;\;\;+0.12231c_{\left(22,23,4\right)}+0.44616s_{\left(22,23\right)})-0.0002{\dot{\theta }}_{6}(s_{\left(2,3,4,-5\right)}-s_{\left(2,3,4,5\right)}), \end{array}$$

(A37) $$\begin{array}{cc} C_{32}= & {\dot{\theta }}_{2}\left(0.16253(c_{\left(3\right)}c_{\left(4\right)}-s_{\left(3\right)}s_{\left(4\right)})+0.04315(c_{\left(4\right)}s_{\left(3\right)}s_{\left(5\right)}+c_{\left(3\right)}s_{\left(4\right)}s_{\left(5\right)})+1.2249s_{\left(3\right)}\right)\\ & -{\dot{\theta }}_{4}\left(0.12231c_{\left(4\right)}+0.03247s_{\left(4\right)}s_{\left(5\right)}\right)+{\dot{\theta }}_{5}\left(0.00288s_{\left(25\right)}+0.03247c_{\left(4\right)}c_{\left(5\right)}\right), \end{array}$$

(A38) $$C_{33}={\dot{\theta }}_{5}\left(0.03247c_{\left(4\right)}c_{\left(5\right)}+0.00576c_{\left(5\right)}s_{\left(5\right)}\right)-{\dot{\theta }}_{4}\left(0.12231c_{\left(4\right)}+0.03247s_{\left(4\right)}s_{\left(5\right)}\right),$$

(A39) $$\begin{array}{cc} C_{34}= & {\dot{\theta }}_{5}\left(0.03247c_{\left(4\right)}c_{\left(5\right)}+0.00576c_{\left(5\right)}s_{\left(5\right)}\right)-{\dot{\theta }}_{4}(0.12231c_{\left(4\right)}+0.03247s_{\left(4\right)}s_{\left(5\right)})-{\dot{\theta }}_{3}(0.12231c_{\left(4\right)}\\ & \;\;\;\;\;\;\;\;+0.03247s_{\left(4\right)}s_{\left(5\right)})-{\dot{\theta }}_{2}(0.12231c_{\left(4\right)}+0.03247s_{\left(4\right)}s_{\left(5\right)}), \end{array}$$

(A40) $$\begin{array}{cc} C_{35}= & {\dot{\theta }}_{1}(0.01623(c_{\left(2,3,5\right)}-c_{\left(2,3,-5\right)})+0.00144(s_{\left(2,3,4,-25\right)}-s_{\left(2,3,4,25\right)})+0.00493(s_{\left(2,3,4,-5\right)}+s_{\left(2,3,4,5\right)})\\ & \;\;\;\;\;\;\;\;\;\;-0.00423s_{\left(2,3,4\right)})+{\dot{\theta }}_{3}(0.01623(c_{\left(4,-5\right)}+c_{\left(4,5\right)})+0.00288s_{\left(25\right)})+{\dot{\theta }}_{4}(0.01623(c_{\left(4,-5\right)}\\ & \;\;\;\;\;\;\;\;\;\;+c_{\left(4,5\right)})+0.00288s_{\left(25\right)})-0.0004{\dot{\theta }}_{6}s_{\left(5\right)}+{\dot{\theta }}_{5}(0.01623(c_{\left(4,5\right)}-c_{\left(4,-5\right)})+0.00987s_{\left(5\right)})\\ & \;\;\;\;\;\;\;\;\;\;+{\dot{\theta }}_{2}(0.01623(c_{\left(4,5\right)}+c_{\left(4,-5\right)})+0.00288s_{\left(25\right)}), \end{array}$$

(A41) $$\begin{array}{cc} C_{41}= & {\dot{\theta }}_{5}\left(0.00493(s_{\left(2,3,4,-5\right)}-s_{\left(2,3,4,5\right)})+0.00144(s_{\left(2,3,4,-25\right)}+s_{\left(2,3,4,25\right)})-0.00423s_{\left(2,3,4\right)}\right)\\ & \;\;+{\dot{\theta }}_{1}(0.00811(c_{\left(4,-5\right)}+c_{\left(22,23,4,-5\right)}-c_{\left(4,5\right)}-c_{\left(22,23,4,5\right)})+0.00493(s_{\left(22,23,24,5\right)}\\ & \;\;-s_{\left(22,23,24,-5\right)})-0.00072\left(s_{\left(22,23,24,25\right)}+s_{\left(22,23,24,-25\right)}\right)-0.01078(c_{\left(3,4,5\right)}+c_{\left(22,3,4,5\right)}\\ & \;\;-c_{\left(3,4,-5\right)}-c_{\left(22,3,4,-5\right)})+0.06115(c_{\left(22,23,4\right)}+c_{\left(4\right)})+0.08126(c_{\left(3,4\right)}+c_{\left(22,3,4\right)})\\ & \;\;-0.01674s_{\left(22,23,24\right)})-0.0002{\dot{\theta }}_{6}(s_{\left(2,3,4,-5\right)}-s_{\left(2,3,4,5\right)}), \end{array}$$

(A42) $$\begin{array}{cc} C_{42}= & {\dot{\theta }}_{3}(0.12231c_{\left(4\right)}+0.03247s_{\left(4\right)}s_{\left(5\right)})+0.00288{\dot{\theta }}_{5}c_{\left(25\right)}+{\dot{\theta }}_{2}(0.16253\left(c_{\left(3\right)}c_{\left(4\right)}-s_{\left(3\right)}s_{\left(4\right)}\right)\\ & \;\;+0.04315(c_{\left(4\right)}s_{\left(3\right)}s_{\left(5\right)}+c_{\left(3\right)}s_{\left(4\right)}s_{\left(5\right)})+0.12231c_{\left(4\right)}+0.03247s_{\left(4\right)}s_{\left(5\right)}, \end{array}$$

(A43) $$C_{43}={\dot{\theta }}_{2}\left(0.12231c_{\left(4\right)}+0.03247s_{\left(3\right)}s_{\left(4\right)}\right)+{\dot{\theta }}_{3}\left(0.12231c_{\left(4\right)}+0.03247s_{\left(4\right)}s_{\left(5\right)}\right)+0.00576{\dot{\theta }}_{5}s_{\left(5\right)}c_{\left(5\right)},$$

(A44) $$C_{44}=0.00288{\dot{\theta }}_{5}s_{\left(25\right)},$$

(A45) $$\begin{array}{cc} C_{45}= & {\dot{\theta }}_{1}\left(0.00493(s_{\left(2,3,4,-5\right)}-s_{\left(2,3,4,5\right)})-0.00423s_{\left(2,3,4\right)}+0.00144(s_{\left(2,3,4,-25\right)}+s_{\left(2,3,4,25\right)})\right)\\ & \;\;+0.00288{\dot{\theta }}_{2}s_{\left(25\right)}+0.00288{\dot{\theta }}_{4}s_{\left(25\right)}+0.00987{\dot{\theta }}_{5}s_{\left(5\right)}-0.0004{\dot{\theta }}_{6}s_{\left(5\right)}+0.00288{\dot{\theta }}_{3}s_{\left(25\right)}, \end{array}$$

(A46) $$\begin{array}{cc} C_{51}= & 0.0002{\dot{\theta }}_{6}(s_{\left(2,3,4,-5\right)}+s_{\left(2,3,4,5\right)})-{\dot{\theta }}_{3}(0.01623(c_{\left(2,3,5\right)}-c_{\left(2,3,-5\right)})+0.00493(s_{\left(2,3,4,-5\right)}-s_{\left(2,3,4,5\right)})\\ & \;\;\;\;\;\;\;\;\;+0.00144(s_{\left(2,3,4,-25\right)}+s_{\left(2,3,4,25\right)}))-{\dot{\theta }}_{2}(0.01623(c_{\left(2,3,5\right)}-c_{\left(2,3,-5\right)})+0.00493(s_{\left(2,3,4,-5\right)}\\ & \;\;\;\;\;\;\;\;\;-s_{\left(2,3,4,5\right)})+0.02157(c_{\left(2,5\right)}-c_{\left(2,-5\right)})+0.00144(s_{\left(2,3,4,-25\right)}+s_{\left(2,3,4,25\right)})-0.00423s_{\left(2,3,4\right)})\\ & \;\;\;\;\;\;\;\;\;-{\dot{\theta }}_{4}(0.00493(s_{\left(2,3,4,-5\right)}-s_{\left(2,3,4,5\right)})+0.00144(s_{\left(2,3,4,-25\right)}+s_{\left(2,3,4,25\right)})-0.00423s_{\left(2,3,4\right)})\\ & \;\;\;\;\;\;\;\;\;-{\dot{\theta }}_{1}(0.00811(c_{\left(4,-5\right)}+c_{\left(22,23,4,-5\right)}+c_{\left(4,5\right)}+c_{\left(22,23,4,5\right)})\\ & \;\;\;\;\;\;\;\;\;+0.00072(s_{\left(22,23,24,25\right)}{-s}_{\left(22,23,24,-25\right)})-0.00246(s_{\left(22,23,24,5\right)}+s_{\left(22,23,24,-5\right)})\\ & \;\;\;\;\;\;\;\;\;+0.01078(c_{\left(3,4,5\right)}+c_{\left(22,3,4,5\right)}+c_{\left(3,4,-5\right)}+c_{\left(22,3,45\right)})+-0.00144s_{\left(25\right)}-0.01570s_{\left(5\right)}), \end{array}$$

(A47) $$\begin{array}{cc} C_{52}= & 0.0004{\dot{\theta }}_{6}s_{\left(5\right)}-{\dot{\theta }}_{1}(0.01623(c_{\left(2,3,5\right)}-c_{\left(2,3,-5\right)})+0.00493(s_{\left(2,3,4,-5\right)}-s_{\left(2,3,4,5\right)})+0.02157(c_{\left(2,5\right)}\\ & \;\;\;\;\;\;\;\;-c_{\left(2,-5\right)})+0.00144(s_{\left(2,3,4,-25\right)}+s_{\left(2,3,4,25\right)})-0.00423s_{\left(2,3,4\right)})+-0.00288{\dot{\theta }}_{4}s_{\left(25\right)}\\ & \;\;\;\;\;\;\;\;-{\dot{\theta }}_{2}(0.02157(c_{\left(3,4,5\right)}+c_{\left(3,4,-5\right)})+0.01623(c_{\left(4,-5\right)}+c_{\left(4,5\right)})+0.00288s_{\left(25\right)})\\ & \;\;\;\;\;\;\;\;-{\dot{\theta }}_{3}(0.01623(c_{\left(4,-5\right)}+c_{\left(4,5\right)})+0.00288s_{\left(25\right)}), \end{array}$$

(A48) $$\begin{array}{cc} C_{53}= & 0.0004{\dot{\theta }}_{6}s_{\left(5\right)}-{\dot{\theta }}_{3}(0.01623(c_{\left(4,-5\right)}+c_{\left(4,5\right)})+0.00288s_{\left(25\right)})-{\dot{\theta }}_{1}(0.01623(c_{\left(2,3,5\right)}-c_{\left(2,3,-5\right)})\\ & \;\;\;\;\;\;\;+0.00493(s_{\left(2,3,4,-5\right)}-s_{\left(2,3,4,5\right)})+0.00144(s_{\left(2,3,4,-25\right)}+s_{\left(2,3,4,25\right)})-0.00423s_{\left(2,3,4\right)})\\ & \;\;\;\;\;\;\;-0.00288{\dot{\theta }}_{4}s_{\left(25\right)}-{\dot{\theta }}_{2}(+0.01623(c_{\left(4,-5\right)}+c_{\left(4,5\right)})+0.00288s_{\left(25\right)}), \end{array}$$

(A49) $$\begin{array}{cc} C_{54}= & 0.0004{\dot{\theta }}_{6}s_{\left(5\right)}-{\dot{\theta }}_{3}0.00288s_{\left(25\right)})-{\dot{\theta }}_{1}(0.00493(s_{\left(2,3,4,-5\right)}-s_{\left(2,3,4,5\right)})+0.00144(s_{\left(2,3,4,-25\right)}+s_{\left(2,3,4,25\right)})\\ & \;\;\;\;\;\;\;\;\;\;\;\;\;\;-0.00423s_{\left(2,3,4\right)})-0.00288{\dot{\theta }}_{4}s_{\left(25\right)}-0.00288{\dot{\theta }}_{2}s_{\left(25\right)}, \end{array}$$

(A50) $$C_{56}=0.0002{\dot{\theta }}_{1}\left(s_{\left(2,3,4,-5\right)}-s_{\left(2,3,4,5\right)}\right)+0.0004s_{\left(5\right)}({\dot{\theta }}_{2}+{\dot{\theta }}_{3}+{\dot{\theta }}_{4}),$$

(A51) $$C_{65}=-0.0002{\dot{\theta }}_{1}\left(s_{\left(2,3,4,5\right)}+s_{\left(2,3,4,5\right)}\right)-0.0004s_{\left(5\right)}({\dot{\theta }}_{2}+{\dot{\theta }}_{3}+{\dot{\theta }}_{4}).$$

(A52) $$G={\left[\begin{array}{cccccc} G_{11} & G_{21} & G_{31} & G_{41} & G_{51} & G_{61} \end{array}\right]}^{T},$$

(A53) $$G_{11}=G_{61}=0,$$

(A54) $$\begin{array}{cc} G_{21}= & 62.39c_{\left(2\right)}+25.12c_{\left(2\right)}c_{\left(3\right)}-3.333s_{\left(4\right)}\left(c_{\left(2\right)}c_{\left(3\right)}-s_{\left(2\right)}s_{\left(3\right)}\right)-25.12s_{\left(2\right)}s_{\left(3\right)}\\ & \;\;\;\;\;\;\;\;\;\;\;\;\;\;\;\;+0.8849s_{\left(5\right)}\left(c_{\left(4\right)}\left(c_{\left(2\right)}c_{\left(3\right)}-s_{\left(2\right)}s_{\left(3\right)}\right)\right.\left.-s_{\left(4\right)}\left(c_{\left(2\right)}s_{\left(3\right)}+c_{\left(3\right)}s_{\left(2\right)}\right)\right)\\ & \;\;\;\;\;\;\;\;\;\;\;\;\;\;\;\;-3.333c_{\left(4\right)}\left(c_{\left(2\right)}s_{\left(3\right)}+c_{\left(3\right)}s_{\left(2\right)}\right), \end{array}$$

(A55) $$\begin{array}{cc} G_{31}= & 25.12c_{\left(2\right)}c_{\left(3\right)}-3.333s_{\left(4\right)}\left(c_{\left(2\right)}c_{\left(3\right)}-s_{\left(2\right)}s_{\left(3\right)}\right)-25.12s_{\left(2\right)}s_{\left(3\right)}+0.8849s_{\left(5\right)}(c_{\left(4\right)}(c_{\left(2\right)}c_{\left(3\right)}-s_{\left(2\right)}s_{\left(3\right)}))\\ & \;\;\;\;\;\;\;\;\;\;-s_{\left(4\right)}\left(c_{\left(2\right)}s_{\left(3\right)}\right.\left.c_{\left(3\right)}s_{\left(2\right)}\right))-3.333c_{\left(4\right)}\left(c_{\left(2\right)}s_{\left(3\right)}-s_{\left(2\right)}c_{\left(3\right)}\right), \end{array}$$

(A56) $$\begin{array}{cc} G_{41}= & 0.8849s_{\left(5\right)}\left(c_{\left(4\right)}\left(c_{\left(2\right)}c_{\left(3\right)}-s_{\left(2\right)}s_{\left(3\right)}\right)-s_{\left(4\right)}\left(c_{\left(2\right)}s_{\left(3\right)}+c_{\left(3\right)}s_{\left(2\right)}\right)\right)-3.333s_{\left(4\right)}\left(c_{\left(2\right)}c_{\left(3\right)}-s_{\left(2\right)}s_{\left(3\right)}\right)\\ & \;\;\;\;\;\;\;-3.333c_{\left(4\right)}\left(c_{\left(2\right)}s_{\left(3\right)}-s_{\left(2\right)}c_{\left(3\right)}\right), \end{array}$$

(A57) $$G_{51}=0.8849c_{\left(5\right)}\left(s_{\left(4\right)}\left(c_{\left(2\right)}c_{\left(3\right)}-s_{\left(2\right)}s_{\left(3\right)}\right)+c_{\left(4\right)}\left(c_{\left(2\right)}s_{\left(3\right)}+c_{\left(3\right)}s_{\left(2\right)}\right)\right).$$
